# One-dimensional Bi–Br pyrazinamide-based hybrid halide perovskite: crystal architecture, intermolecular interactions, and structure–optical properties relationship

**DOI:** 10.1039/d6ra08608j

**Published:** 2026-11-10

**Authors:** Amin Alibi, Nour Elleuch, Emna Khdhiri, Sergiu Shova, Jerome Lhoste, Houcine Ammar, Mohamed Boujelbene

**Affiliations:** a Laboratory of Physico-Chemistry of Solid State, LR11ES51, Sfax Faculty of Sciences, University of Sfax Sfax 3000 Tunisia; b Laboratory of Chemistry, Heterocycles, Coprs Gras and Polymers, Sfax Faculty of Sciences, University of Sfax Sfax 3000 Tunisia; c “Petru Poni” Institute of Macromolecular Chemistry Alea Grigore Ghica voda 41-A 700487 Iasi Romania; d MMM-UMR 6283 CNRS, Lunam, Faculty of Sciences and Techniques, University of Maine Avenue Olivier Messiaen, 72085. Le Mans Cedex 9 France

## Abstract

A one-dimensional bismuth bromide organic–inorganic hybrid halide perovskite based on protonated pyrazinamide cations, (C_5_H_6_N_3_O)_2_·[BiBr_5_], is reported and comprehensively investigated. Single-crystal X-ray diffraction reveals a perovskite-related architecture built from corner-sharing [BiBr_6_] octahedra forming one-dimensional chains, stabilized by a dense network of N–H⋯Br and N–H⋯O hydrogen bonds together with significant π–π stacking interactions. Hirshfeld surface analysis quantitatively confirms the dominance of halogen-mediated hydrogen bonding in governing the supramolecular organization. Diffuse reflectance spectroscopy establishes the material as a direct-band-gap semiconductor with an optical gap of ∼2.8 eV, supported by a sharp absorption edge, low Urbach energy, and consistent threshold wavelength analysis. Photoluminescence measurements reveal a single intense phosphorescent emission centered at 540 nm, with CIE coordinates located in the yellowish-green region. Density functional theory calculations, including DOS, PDOS, ELF, LOL, and NCI–RDG analyses, provide microscopic insight into the electronic structure and intermolecular interactions, highlighting charge-transfer contributions from the inorganic sublattice to the organic moiety near the band edges. The combined results establish clear structure–property correlations in this lead-free Bi-based hybrid system and underline its relevance within the broader family of perovskite-inspired semiconductors.

## Abstract 2

A one-dimensional bismuth bromide organic–inorganic hybrid halide perovskite based on protonated pyrazinamide cations, (C_5_H_6_N_3_O)_2_·[BiBr_5_], is reported and comprehensively investigated.

## Graphical abstract

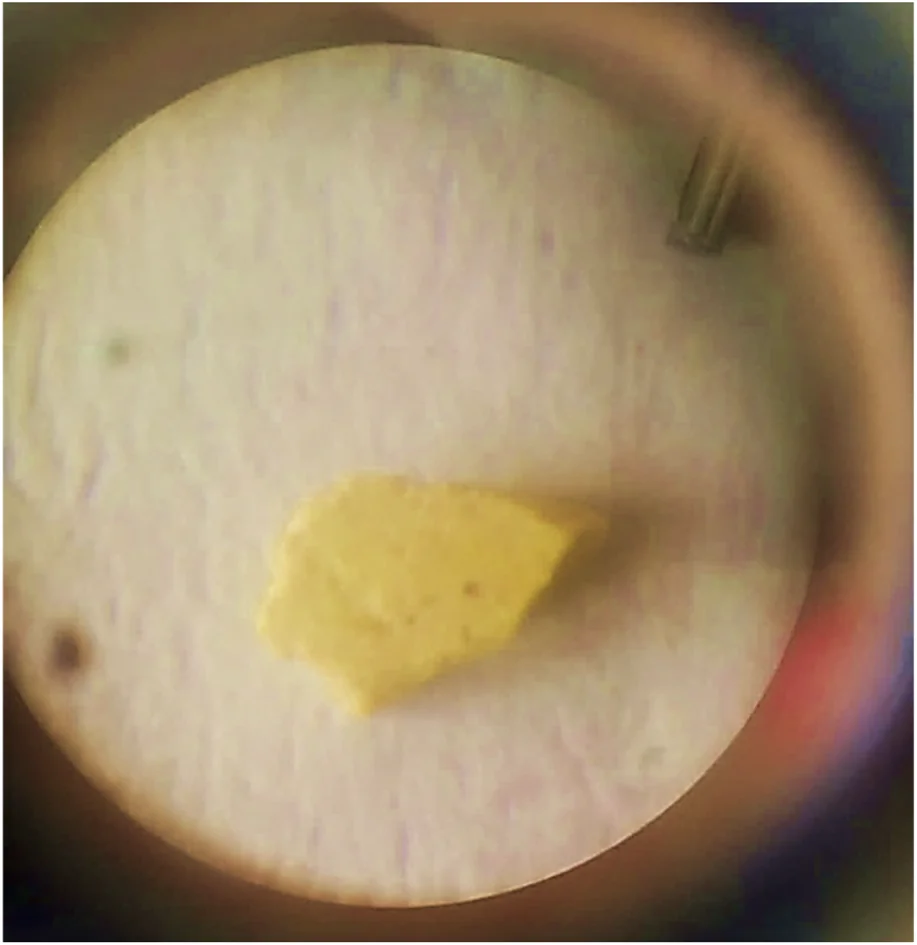

## 1. Introduction

Organic–inorganic hybrid halide materials based on heavy p-block elements have attracted growing attention due to their structural diversity and tunable physical properties.^1^ Among them, bismuth-based hybrid halometalates have emerged as attractive alternatives to lead-containing systems, combining improved chemical stability and reduced toxicity with promising optical characteristics. These advantages have motivated extensive efforts to elucidate their structure–property relationships and to evaluate their potential in solar energy utilization,^2–5^ nonlinear optical devices,^6,7^ light-emitting diodes,^8,9^ photoconductors,^10^ optical microcavities,^11^ and thin-film field-effect transistors.^12^

Bismuth shares key electronic features with lead, notably the presence of a stereochemically active 6s^2^ lone pair, which strongly influences coordination geometry, octahedral distortion, and electronic structure. Unlike lead, however, bismuth exhibits significantly lower biological toxicity due to the limited solubility of most of its compounds. Despite these advantages, bismuth halometalates frequently exhibit modest optoelectronic performance, particularly with respect to charge transport efficiency, underscoring the importance of understanding how structural dimensionality and intermolecular interactions govern their optical response.^13^

Halobismuthates display remarkable structural flexibility, ranging from zero-dimensional clusters to one- and two-dimensional inorganic frameworks,^14,15^ depending on the Bi/halide ratio and the nature of the organic counterions. In low-dimensional systems, especially one-dimensional architectures, the electronic properties are highly sensitive to octahedral connectivity, the degree of distortion within the BiX_6_ units, and the strength of supramolecular interactions. Hydrogen bonding, halogen contacts, and π–π stacking interactions contribute significantly to structural stabilization and can modulate electronic coupling between inorganic chains and organic moieties.^16,17^ Therefore, the rational selection of multifunctional organic cations represents a key strategy for tuning dimensionality, lattice rigidity, and optoelectronic behavior.

In this context, protonated pyrazinamide was deliberately selected as the organic component due to its multifunctional molecular architecture. The pyrazinamide molecule contains an aromatic heterocyclic ring capable of π-electron delocalization, two ring nitrogen atoms, and an amide functional group, providing multiple hydrogen-bonding sites and directional interaction capabilities. Upon protonation, the cation enhances electrostatic stabilization with the inorganic [BiBr_5_]^2−^ chains while preserving its ability to form strong and directional N–H⋯Br hydrogen bonds. Compared with commonly employed small cations such as methylammonium or formamidinium, protonated pyrazinamide introduces greater molecular size, structural rigidity, and extended supramolecular connectivity. These features are expected to reinforce lattice cohesion, influence octahedral distortion, and potentially affect excitonic confinement and emission behavior in low-dimensional bismuth halide systems.

Herein, we report the synthesis and comprehensive characterization of a new one-dimensional bromobismuthate hybrid, (C_5_H_6_N_3_O)_2_·[BiBr_5_]. Single-crystal X-ray diffraction reveals infinite chains of corner-sharing BiBr_6_ octahedra arranged in a one-dimensional motif and stabilized by a dense hydrogen-bonding network involving the protonated pyrazinamide cations. Hirshfeld surface analysis provides quantitative insight into the intermolecular interactions governing the crystal packing.

The optical properties were investigated by diffuse reflectance spectroscopy and photoluminescence measurements to evaluate the semiconducting behavior and emission characteristics of the compound. Density functional theory calculations complement the experimental results by elucidating the electronic structure and charge distribution, while vibrational spectroscopy confirms the integrity of both inorganic and organic substructures. Thermogravimetric analysis further assesses the thermal stability of the hybrid framework.

Through the combined structural, spectroscopic, and theoretical investigation, this work demonstrates how the deliberate incorporation of a multifunctional aromatic cation can modulate structural organization and optical behavior in one-dimensional bismuth-based hybrid halides, providing insight into the rational design of lead-free hybrid semiconductors with tailored properties.

## 2. Experimental

### 2.1. Materials and characterization techniques

The title hybrid compound was synthesized using commercially available bismuth bromide (BiBr_3_), hydrobromic acid (HBr), and the organic precursor pyrazinamide (C_5_H_5_N_3_O), all employed as received without further purification. Vibrational characterization was carried out to gain detailed insight into the molecular composition and bonding environment of the crystalline material. Fourier-transform infrared (FT-IR) spectra were recorded at room temperature using a PerkinElmer FT-IR Paragon 1000 PC spectrometer over the 4000–500 cm^−1^ range. Complementary Raman scattering measurements were performed at room temperature using a Horiba/Jobin-Yvon T6400 spectrometer, covering the spectral window from 4000 to 50 cm^−1^.

The optical behavior of the compound was investigated in the solid state by UV-vis spectroscopy. Diffuse reflectance spectra were collected at room temperature using a PerkinElmer Lambda 35 UV-vis spectrophotometer equipped with an integrating sphere. Measurements were carried out on a 6 mm diameter pellet over the wavelength range of 200–1100 nm. In addition, photoluminescence (PL) spectra were acquired at room temperature using a PerkinElmer LS 55 spectrometer, enabling the determination of emission features and assessment of radiative recombination processes. To accurately evaluate the optical band gap and establish the nature of the electronic transition, a correlation between solid-state diffuse reflectance data and solution-phase UV-vis absorption spectra was established, further supported by time-dependent density functional theory (TD-DFT) calculations performed for both states.

### 2.2. Synthesis of (C_5_H_6_N_3_O)_2_·[BiBr_5_]

The hybrid compound (C_5_H_6_N_3_O)_2_·[BiBr_5_] was synthesized by slow evaporation under ambient conditions (approximately 28 °C). Pyrazinamide (C_5_H_5_N_3_O, 1.00 mmol, 0.1231 g) and bismuth bromide (BiBr_3_, 1.00 mmol; 98% purity) were dissolved separately in 10 mL of distilled water each, corresponding to nominal solution concentrations of 0.10 M. The two solutions were then combined, and 2 mL of concentrated hydrobromic acid (HBr, 48 wt%) was added dropwise in three equal portions at 30 min intervals under continuous stirring. Following the final addition, the mixture was stirred for an additional 30 min, giving a total stirring time of 1.5 h. The resulting solution was left to evaporate slowly for four days at approximately 28 °C, yielding yellow prismatic crystals (Fig. 1). The crystals were collected by filtration, and a suitable single crystal was selected for single-crystal X-ray diffraction analysis. The phase purity of the obtained material was assessed by powder X-ray diffraction. The calculated yield is approximately 80%.

**Fig. 1:**
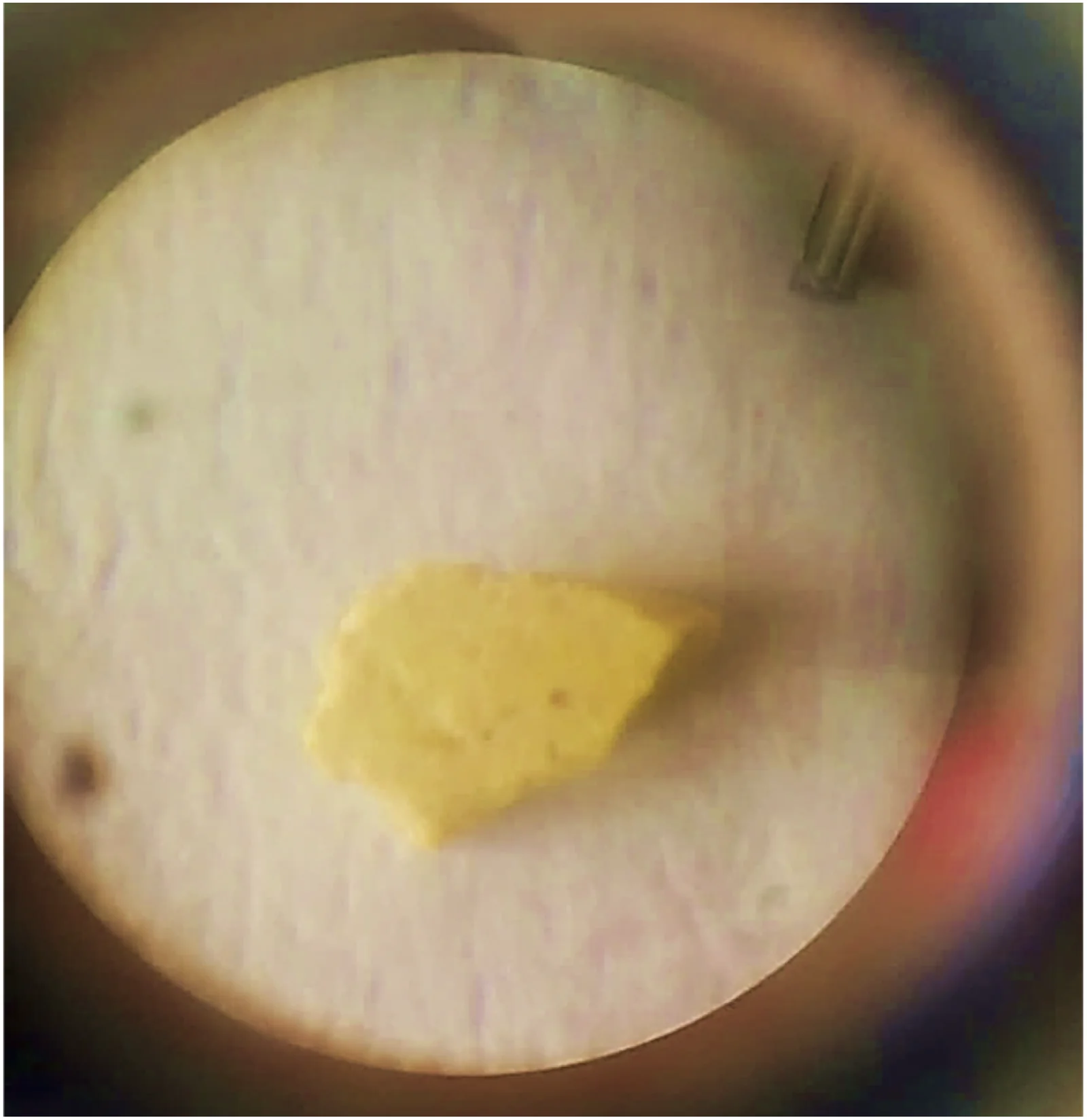
Microscopic image of (C_5_H_6_N_3_O)_2_·[BiBr_5_] crystal.

The purity of the compound was tested using powder XRD, and the results are shown in Fig. S1, showing great agreement between experimental and calculated peaks, demonstrating the purity of the crystals.

### 2.3. Single-crystal X-ray data collection and structure determination

The crystal structure of the hybrid compound (C_5_H_6_N_3_O)_2_·[BiBr_5_] was determined by single-crystal X-ray diffraction at 293 K. Data collection was carried out using Mo Kα radiation (*λ* = 0.71073 Å) on an XtaLAB Synergy Dualflex HyPix diffractometer, employing a carefully selected yellow prismatic crystal with approximate dimensions of 0.15 × 0.15 × 0.05 mm^3^. The crystallographic data were processed and analyzed using the Olex2 (v1.5) software package.^18^ Structure solution was achieved by direct methods and subsequently refined by full-matrix least-squares minimization on *F*^2^ using SHELXT 2018/2 (ref. 19) and SHELXL 2018/3,^20^ respectively.

All non-hydrogen atoms were refined anisotropically, allowing precise determination of their positional and displacement parameters. In contrast to commonly applied riding models, all hydrogen atoms were located directly from the experimental Fourier difference maps and refined isotropically without geometrical constraints (no HFIX restraints). This approach ensured an experimentally reliable description of the hydrogen positions and enabled an accurate characterization of the hydrogen-bonding network involving both the protonated organic cations and the inorganic bromide framework. Graphical representations of the molecular geometry and crystal packing were generated using DIAMOND 3 software.^21^ After successive refinement cycles, the structure converged to final agreement factors of *R*_1_ = 0.039 and w*R*_2_ = 0.068, attesting to the high quality of the crystallographic model. Complete details of the data collection, refinement parameters, and crystallographic metrics are summarized in Table S1.

### 2.4. Hirshfeld surface analysis

Hirshfeld surface analysis^22–25^ was employed to identify, quantify, and visualize the various noncovalent interactions governing the crystal packing,^26,27^ including hydrogen bonding, π–π stacking, and halogen-mediated contacts. This approach enables the partitioning of the crystal electron density into discrete molecular regions, thereby allowing a detailed and molecule-centered examination of intermolecular interactions within the solid state. Structural information obtained from single-crystal X-ray diffraction and provided in CIF format was analyzed using the CrystalExplorer 21.5 software^28^ package to construct Hirshfeld surfaces and their associated two-dimensional fingerprint plots,^29,30^ which provide a quantitative representation of the relative contributions of the different interaction types to the overall crystal packing.

Each point on a Hirshfeld surface is defined by two distances: *d*_i_, corresponding to the distance from the surface to the nearest atomic nucleus inside the surface, and *d*_e_, representing the distance to the closest nucleus external to the surface. These parameters are combined to calculate the normalized contact distance *d*_norm_, which reflects the proximity of intermolecular contacts relative to van der Waals radii and enables the visual identification of close, significant interactions through color mapping on the surface, as defined by the corresponding normalization equations:
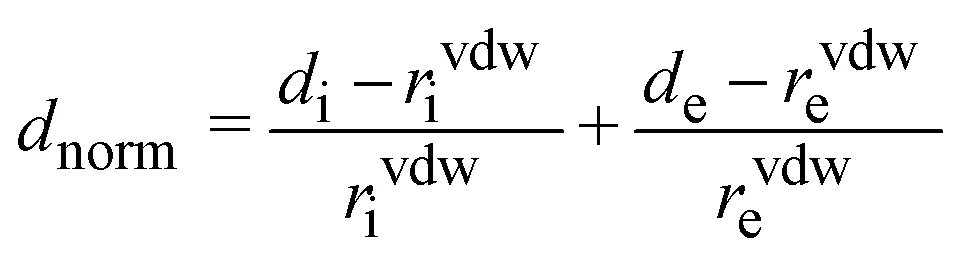
where *r*^vdw^_i_ and *r*^vdw^_e_ are the atoms' van der Waals radii.^31,32^

### 2.5. Thermal analysis

Thermogravimetric analysis (TGA) was performed to assess the thermal stability and decomposition behavior of the hybrid material. Measurements were carried out using a SETA RAM 92 multi-module thermal analyzer under an inert nitrogen atmosphere. The sample (5.763 mg) was heated from room temperature to 380 °C at a constant heating rate of 10 °C min^−1^, allowing detailed monitoring of mass loss events associated with the thermal degradation process.

### 2.6. Computational details

The structural, electronic, and optical properties of the hybrid compound (C_5_H_6_N_3_O)_2_·[BiBr_5_] were examined using density functional theory (DFT). Geometry optimizations, vibrational frequency calculations, and frontier orbital and energy gap analyses were performed with the B3LYP hybrid functional^33^ as implemented in Gaussian 09W,^34^ employing the GENECP scheme for mixed basis sets. Relativistic effects for Bi and Br atoms were treated using the LANL2DZ effective core potential,^35^ while the 6-311++G* basis set was applied to H, C, N, and O atoms to ensure an accurate all-electron description of the light elements. This combination offers a balanced and computationally efficient treatment of the hybrid system.

A finite cluster model corresponding to the crystallographic formula unit, composed of one [BiBr_5_]^2−^ inorganic moiety and two protonated pyrazinamide cations, was fully optimized. This approach was adopted because periodic DFT calculations of the infinite one-dimensional Bi–Br framework would require large supercells and advanced dispersion treatments to capture long-range hydrogen bonding and π–π interactions, which are not computationally practical at the hybrid-functional level. Nevertheless, the selected cluster reliably reproduces the local coordination environment of Bi(iii), the electronic distribution within the Bi–Br framework, and the key cation–anion interactions relevant to the experimental observations.

Harmonic vibrational frequencies were calculated and compared with the experimental FT-IR and Raman spectra, confirming the stability of the optimized structure and supporting the vibrational assignments. Mode visualization was carried out using GaussView 6.0.16.^36^ Electronic structure analyses, including DOS, ELF, LOL, NCI–RDG, and electrostatic potential mapping, were performed using Multiwfn,^37^ providing a consistent interpretation of bonding characteristics, noncovalent interactions, and optical behavior in close agreement with the experimental results.

## 3. Results and discussion

### 3.1. Structural study

The compound (C_5_H_6_N_3_O)_2_·[BiBr_5_] crystallizes in the monoclinic space group *I*2/*a*, with unit-cell parameters *a* = 9.0086(5) Å, *b* = 11.9836(6) Å, *c* = 18.7178(10) Å and *β* = 90.870(2)°. Its asymmetric unit consists of a single monoprotonated pyrazinamide cation and a BiBr_5_ fragment, the latter representing only half of the inorganic octahedron because the bismuth atom lies exactly on a crystallographic twofold rotation axis (*Z*′ = 0.5). In Fig. 2(a), the full formula unit is depicted, highlighting the arrangement of the two pyrazinamidinium cations around the completed [BiBr_6_]^3−^ octahedron. As shown in Fig. 2(b), the inorganic portion present before applying symmetry is not BiBr_6_ but rather a BiBr_3_ “hemioctahedron”, comprising only the three bromide ligands needed to generate the other three *via* the 2-axis operation. Application of this symmetry element reproduces the missing three bromide ligands, thereby completing the BiBr_6_ octahedron, and simultaneously doubles the number of organic cations. This explains why the empirical formula contains BiBr_5_ per asymmetric unit. Yet, the extended structure contains a fully coordinated BiBr_6_ unit: the “sixth” bromide, Br3^i^, is not chemically absent, but is generated crystallographically rather than independently present in the asymmetric unit. Fig. 2(c) illustrates the exact orientation of the 2-axis relative to the metal center and the halide positions, emphasizing how this axis enforces equivalence of opposing Bi–Br bonds and governs the local geometry of the inorganic core. In Fig. 2(d), the twofold axis is shown within the formula unit itself, clarifying how this symmetry operation organizes both the inorganic anion and the pair of organic cations into a strictly defined structural motif.

**Fig. 2:**
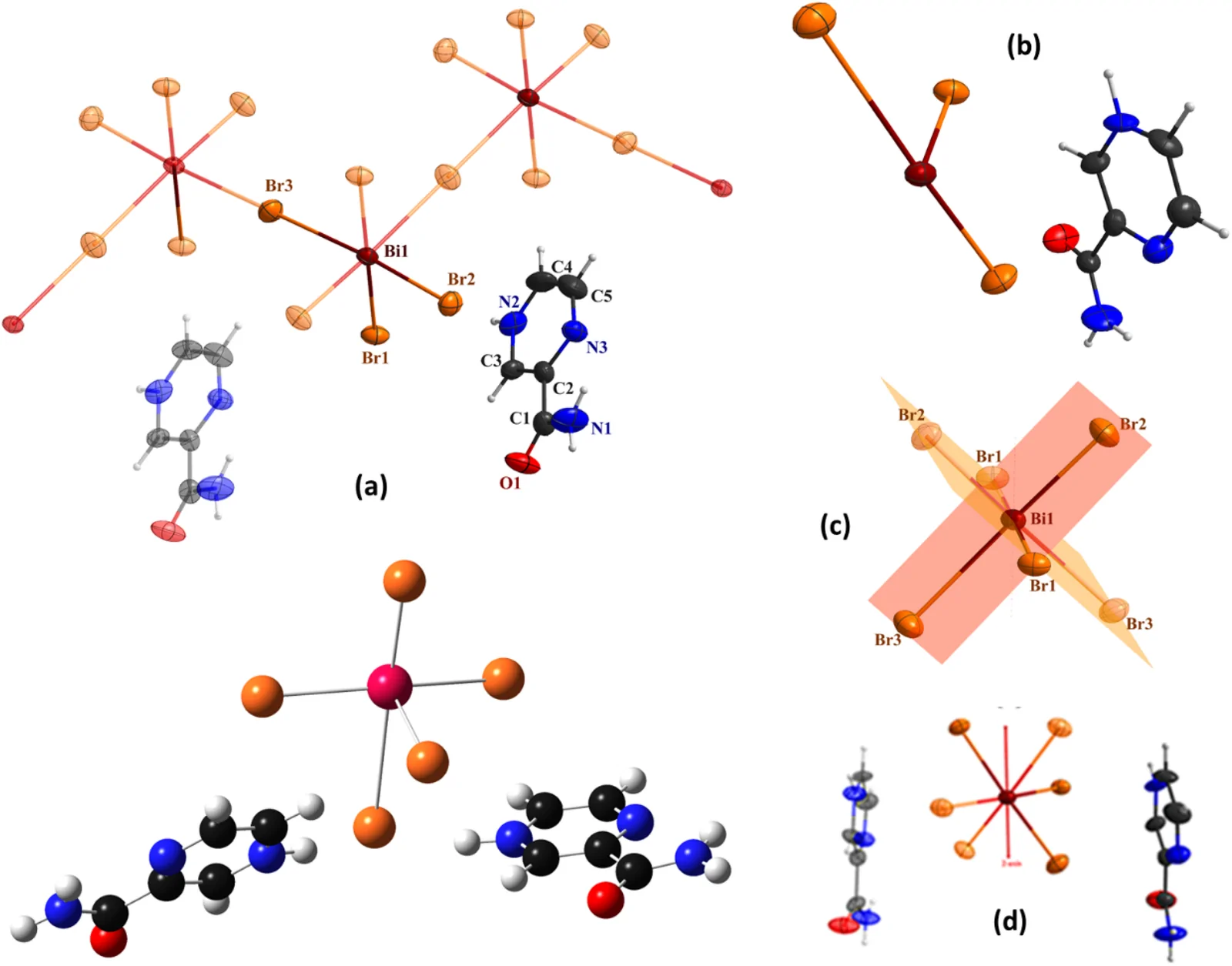
(a) (down) Theoretical and (up) experimental formula units and crystallographic symmetry of (C_5_H_6_N_3_O)_2_·[BiBr_5_] (b) experimental asymmetric unit containing one protonated pyrazinamide cation and a BiBr_3_ hemioctahedron; (c) completion of the BiBr_6_ octahedron through the crystallographic twofold rotation axis; and (d) symmetry-related arrangement of the two organic cations around the inorganic unit.

The protonation state of pyrazinamide was assessed using complementary crystallographic and computational evidence. In the SCXRD refinement, the hydrogen atoms were located from the experimental Fourier difference maps and refined isotropically without geometrical constraints, allowing their positions to be determined directly from the diffraction data. In addition, calculations comparing the basicities of the three nitrogen sites support the experimentally assigned protonation site. Together, these results support the proposed monoprotonated pyrazinamide cation and its role in the hydrogen-bonding network that stabilizes the crystal structure.

Around the octahedral core, the pyrazinamidinium cations adopt orientations dictated by electrostatic complementarity and N–H⋯Br/N–H⋯O hydrogen bonding, preorganizing the supramolecular environment without yet defining long-range connectivity. These symmetry-generated local features constitute the elementary building unit from which the extended one-dimensional architecture develops (Fig. 3). Fig. 3 presents the three-dimensional projection of the unit cell and illustrates how the discrete units shown in Fig. 2 assemble into a continuous inorganic chain running along the *a*-axis. Within the monoclinic *I*2/*a* symmetry, the Bi-centered octahedra align through a linear Br3⋯Bi⋯Br3 bridging motif, where Br3 acts simultaneously as a terminal ligand and, *via* symmetry, as a *trans*-related bridging ligand to the adjacent Bi(iii) center. This corner-sharing connectivity generates an infinite chain of BiBr_6_ octahedra, defining the one-dimensional character of the structure. The twofold axis passing through Bi not only completes the octahedral coordination sphere but also governs the relative orientation of neighboring octahedra along the chain. Symmetry-imposed equivalence of Bi–Br environments ensures uniform octahedral alignment and produces a crystallographically ordered inorganic backbone extending along a. This propagation mode classifies the compound as a one-dimensional halometallic perovskite derivative, where dimensionality directly arises from intrinsic BiBr_6_ connectivity. The pyrazinamidinium cations surround the inorganic column without perturbing the halide framework, acting as charge-compensating and spacing units. Their hydrogen-bond-directed orientations stabilize the lattice and fill the interstitial volume, resulting in an organized, non-interpenetrating packing arrangement. The absence of organic–inorganic covalent bonding preserves the strict topological integrity of the 1D Bi–Br chain.

**Fig. 3:**
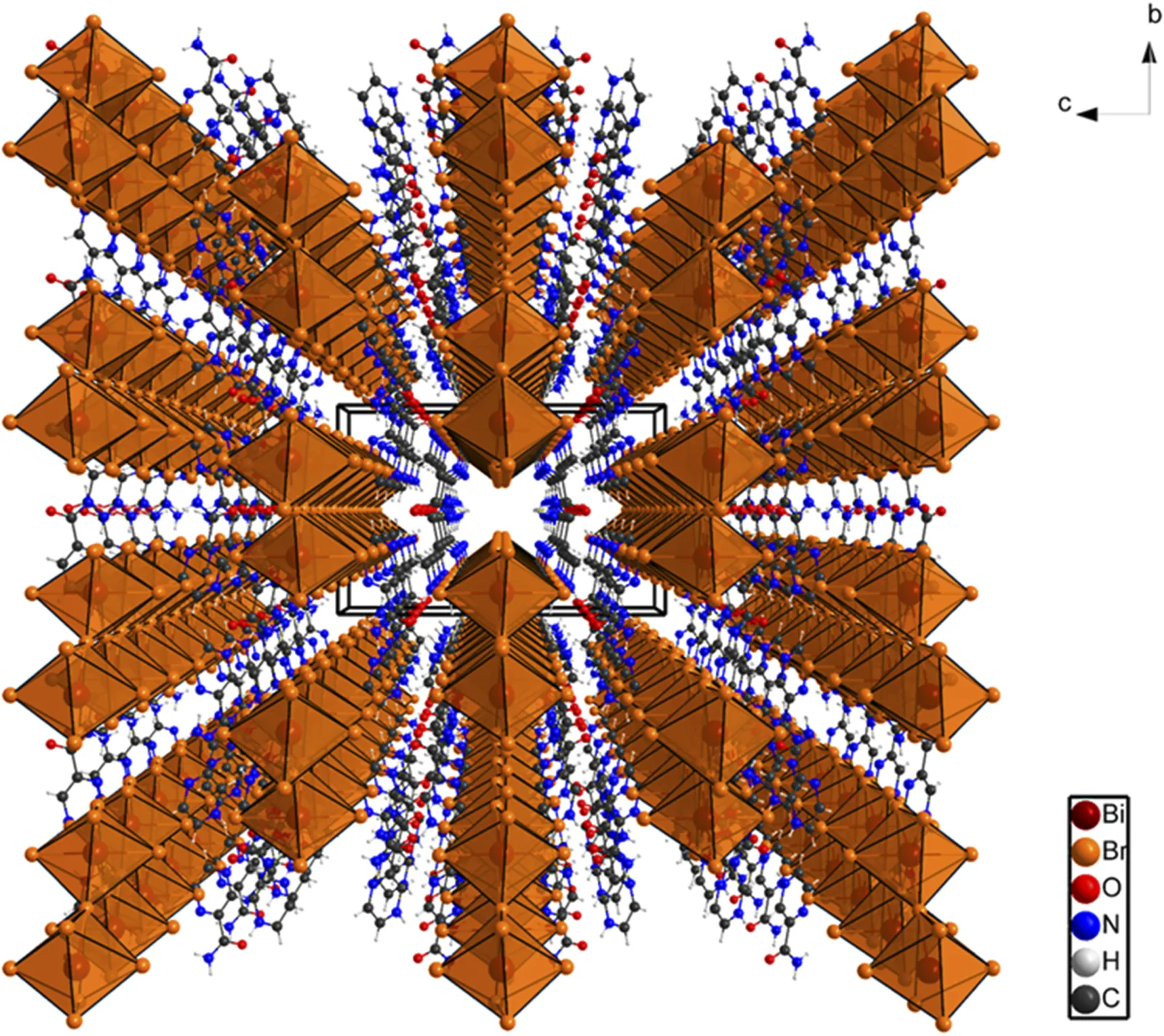
Three-dimensional projection of the unit cell and formation of the one-dimensional chain.

Fig. 4 highlights the contribution of halogen–halogen interactions to the supramolecular organization. Although the inorganic framework is intrinsically one-dimensional, defined by the Bi–Br corner-sharing chain along the *a*-axis, the bromine atoms participate in a single, repetitive Br⋯Br contact of 3.931 Å confined within the (*a*, *b*) planes. As shown in the right-hand projection, this unique interaction repeats uniformly along the chain, generating consistent intraplanar cohesion. The Br⋯Br distance falls within the range characteristic of attractive type-I halogen interactions and reinforces lateral stabilization without disrupting the primary Bi–Br connectivity. Importantly, these halogen contacts do not extend between adjacent planes. The stacked (*a*, *b*) layers are separated along the *c*-axis by distances and orientations that preclude interplanar Br⋯Br interactions. This confinement introduces structural anisotropy: the BiBr_6_ octahedra define a dominant one-dimensional inorganic backbone, while the repeated intraplanar halogen contacts create cooperative lateral packing within the (*a*, *b*) directions. The absence of interactions along *c* preserves the isolation of the 1D chains in that dimension.

**Fig. 4:**
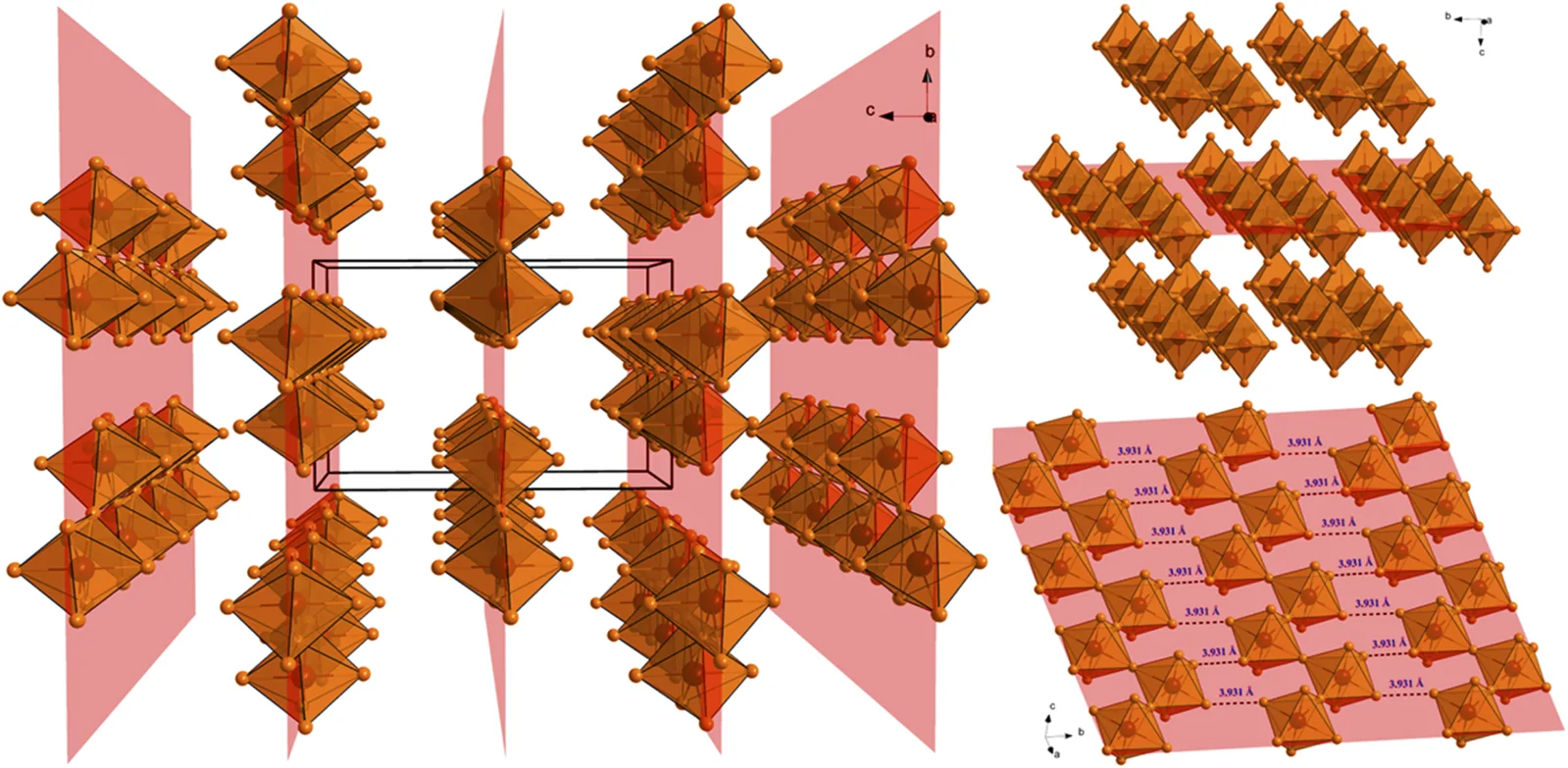
Intraplanar halogen–halogen contacts and emergence of a pseudo-2D structural motif.

Consequently, the structure is best described as a 1D inorganic framework enveloped by a 2D supramolecular halogen-linked network. The Br⋯Br interactions provide planar stabilization and layered organization without true interlayer connectivity, yielding an intermediate dimensionality that is relevant for comparison with related Bi- and Sb-based halide hybrids.

Fig. S2 provides a polyhedral perspective of the BiBr_6_ environment and its supramolecular organization. Within the one-dimensional inorganic backbone, the octahedra are linked exclusively through the Br3 atom, such that each unit shares a single bromide ligand with its neighbor. This single-bridge connectivity defines the chain topology and, under the *I*2/*a* symmetry, imposes a uniform octahedral orientation, yielding a coherent yet minimally constrained 1D framework. The polyhedral representation emphasizes subtle angular deviations arising from the distinct roles of the bridging Br3 *versus* terminal Br1 and Br2 atoms, which govern directional chain propagation and precondition the geometry for secondary supramolecular interactions.

A key feature highlighted in Fig. S2 is the presence of halogen–chalcogen contacts between the oxygen atom of the pyrazinamidinium cation and terminal bromide ligands. Although weaker than classical hydrogen bonds, these directional interactions modulate the local electron density around Br and reinforce the planar stabilization previously associated with Br⋯Br contacts. In parallel, π–π stacking between adjacent pyrazinamide rings consolidates the organic sublattice. These cooperative interactions, propagating in directions complementary to the inorganic chain, generate a rigid supramolecular envelope that counterbalances the intrinsic one-dimensionality of the Bi–Br backbone and enhances overall packing cohesion.

From a comparative standpoint, the chain topology differs markedly from that of (C_10_H_13_N_4_)[BiBr_4_]·2H_2_O,^38^ where octahedra are connected through two bridging halides, producing a denser and more constrained 1D geometry. In contrast, the present structure, crystallizing in *I*2/*a* rather than *P*2_1_/*n*, features single-halide bridging, resulting in a more linear and structurally open chain that is more susceptible to supramolecular modulation. Thus, the observed geometry directly reflects the influence of crystallographic symmetry on dimensionality and connectivity.

Finally, comparison with the SbCl_5_-based hybrid,^39^ in which pyrazinamide undergoes hydrolysis to pyrazinoic acid, underscores the structural fidelity of the present system. Here, the intact monoprotonated pyrazinamide enables π-stacking and halogen–chalcogen interactions that would otherwise be suppressed by chemical transformation. This stability highlights the suitability of pyrazinamide for directing and stabilizing BiBr-based 1D polyhedral chains.

Fig. S3 presents the complete hydrogen-bonding network stabilizing the structure, distinguishing between the extended supramolecular connectivity across the unit cell (left panel) and the minimal interaction motif within a single structural fragment prior to symmetry propagation (right panel). The full list of hydrogen bonds, including distances and angles, is provided in Table 1.

**Table 1:** Interatomic distances and hydrogen bond angles of the compound^a^

D–H⋯A	D–H (Å)	H⋯A (Å)	D⋯A (Å)	D–H⋯A (°)
N1–H1A⋯Br1^iii^	1.01	2.47	3.465 (7)	170
N1–H1B⋯N3	1.01	2.27	2.692 (10)	104
N2–H2⋯Br1	1.03	3.21	3.485 (7)	97
N2–H2⋯O1^iv^	1.03	1.66	2.675 (8)	168
C3–H3⋯Br1	1.08	3.29	3.534 (8)	94
C3–H3⋯O1	1.08	2.47	2.761 (10)	94
C4–H4⋯Br3i	1.08	3.17	3.590 (8)	104
C4–H4⋯O1v	1.08	3.30	3.506 (10)	92

a Symmetry codes: (i) −*x* + 1/2, *y*, −*z* + 1; (iii) *x* + 1/2, −*y* + 1, *z*; (iv) −*x* + 1, *y* + 1/2, −*z* + 1/2; (v) −*x* + 3/2, −*y* + 3/2, −*z* + 1/2.

Four N–H donors participate in the hydrogen-bonding scheme, though their structural roles differ in strength and function. According to Blessing's criterion,^40^ which classifies strong hydrogen bonds near ∼2.7 Å donor–acceptor separation, two interactions are clearly strong:

N1–H1B⋯N3, an intrafragment N–H⋯N contact that stabilizes the conformation of the pyrazinamidinium cation and fixes its orientation within the lattice.

N2–H2⋯O1, a short and highly directional N–H⋯O interaction that reinforces cohesion between neighboring organic units.

The remaining interactions, N1–H1A⋯Br1 and N2–H2⋯Br1, correspond to weak hydrogen bonds following Brown's criterion,^41^ characterized by longer donor–acceptor distances and reduced angular linearity. These contacts provide secondary organic–inorganic linkage without imposing strict geometric constraints.

At the unit-cell level, this network operates hierarchically. The strong N–H⋯N and N–H⋯O interactions rigidify and organize the organic framework, while the weaker N–H⋯Br contacts mediate organic–inorganic integration and assist in consolidating adjacent chains. Because halogen–halogen interactions are confined to the (*a*, *b*) planes and do not propagate along *c*, hydrogen bonding becomes the primary mechanism ensuring interplanar cohesion and three-dimensional consolidation.

In addition to the principal N–H⋯Br and N–H⋯O hydrogen-bonding interactions, the crystallographic geometry identifies several nonconventional C–H⋯Br contacts. Among these, C3–H3⋯Br1 exhibits an H⋯Br distance of 3.29 Å, a donor–acceptor distance of 3.534(8) Å and an angle of 94°, while C4–H4⋯Br3^i^ displays corresponding distances of 3.17 and 3.590(8) Å and an angle of 104°. The relatively long H⋯Br distances and markedly bent geometries suggest that these contacts are weak and less directional than the principal hydrogen bonds. They may nevertheless contribute to the local packing environment and complement the broader network of intermolecular contacts. The overall cohesion of the hybrid structure is therefore primarily associated with the more directional N–H⋯Br and N–H⋯O interactions, together with the other close contacts that organize the organic cations around the one-dimensional Bi–Br framework.

Thus, the structural stability arises from a balanced hierarchy: two strong, highly directional hydrogen bonds governing organic organization, and two weaker N–H⋯Br interactions completing the inorganic–organic integration. Together, they transform the one-dimensional BiBr_6_ chain and pseudo-two-dimensional halogen planes into a rigid and fully interconnected three-dimensional hybrid framework.

As summarized in Table S2, reported one-dimensional bismuth bromide hybrids exhibit diverse crystal architectures and optical responses depending on the nature of the organic cation and the connectivity of the inorganic framework. The present compound, (C_5_H_6_N_3_O)_2_·[BiBr_5_], contributes to this family through the combination of a multifunctional protonated pyrazinamide cation and a one-dimensional framework of corner-sharing BiBr_6_ octahedra. Its crystal packing is further characterized by a network of N–H⋯Br and N–H⋯O hydrogen bonds, together with π–π stacking interactions that contribute to the organization of the organic and inorganic components. The measured optical gap of approximately 2.8 eV, Urbach energy of 0.175 eV, and photoluminescence maximum at 540 nm provide complementary information on its optical response. Unlike studies primarily focused on specific functional properties such as photodetection or ferroelectricity, the present work combines crystallographic analysis, Hirshfeld surface analysis, optical characterization, and computational investigations to establish relationships between crystal architecture, intermolecular interactions, and optical behavior. These complementary characteristics highlight the relevance of the title compound for understanding structure–property relationships in low-dimensional, lead-free bismuth halide hybrids.

### 3.2. Hirshfeld surface analysis (HSA)

Fig. S4 illustrates the Hirshfeld surface analysis of the formula unit of the title compound, providing insight into the intermolecular interactions governing crystal packing. The dnorm-mapped surface exhibits pronounced red regions around the protonated nitrogen atoms of the organic cations, identifying them as the principal hydrogen-bond donors. In contrast, the non-protonated ring nitrogen atoms show fewer pronounced features, indicating their more limited involvement in close intermolecular contacts. Additional red regions occur around the bromide ligands of the one-dimensional Bi–Br chains, highlighting their role as hydrogen-bond acceptors and the importance of N–H⋯Br interactions in connecting the organic and inorganic components.

The shape-index surfaces, presented from both sides of the formula unit, display complementary red and blue triangular patterns on the aromatic rings, characteristic of π–π stacking interactions. These features agree with the crystallographically determined interplanar separation of 3.637 Å, supporting the contribution of aromatic stacking to the organization of the organic sublattice. Furthermore, the curvedness surfaces exhibit extended, relatively flat green regions over the aromatic rings, indicating favorable surface complementarity between adjacent aromatic systems. Together, these descriptors support the coexistence of directional hydrogen bonding and π–π stacking as important interactions contributing to the cohesion of the hybrid structure.

Fig. S5 presents the two-dimensional Hirshfeld fingerprint plots, providing a quantitative assessment of the intermolecular contacts. A total of 15 distinct interaction types are identified, with three categories accounting for the largest contributions. Br⋯H/H⋯Br contacts dominate, representing 49.6% of the total Hirshfeld surface area. These contacts reflect the extensive interactions between bromide ligands and hydrogen atoms of the organic cations. Their fingerprint plot exhibits sharp, symmetrical spikes at short di and de values, consistent with close contacts associated with hydrogen bonding. The O⋯H/H⋯O contacts constitute the second-largest contribution, accounting for 14.8% of the surface. They are associated with interactions involving the amide carbonyl oxygen atoms and are consistent with the N–H⋯O hydrogen bonds identified crystallographically. H⋯H contacts represent a further 9.4%, appearing as a broad, diffuse region along the diagonal of the fingerprint plot. Although generally weak and less directional, these contacts reflect the close packing of the organic moieties and contribute collectively to the overall intermolecular cohesion.

The remaining contact types, including Br⋯Br, C⋯H, N⋯H and C⋯C, account for smaller proportions of the total surface and generally produce less pronounced fingerprint features. Their contributions are secondary to those of the dominant hydrogen-mediated contacts, although they provide additional information about the packing environment. The relatively small contribution from Br⋯Br contacts is consistent with their more localized role in the crystal packing compared with the extensive contacts involving hydrogen atoms. Overall, the fingerprint plots establish a clear hierarchy of intermolecular contacts, highlighting the predominance of hydrogen-mediated interactions in the organization of the organic and inorganic sublattices.

Fig. S6 presents the crystal void analysis, providing quantitative information on the packing efficiency of the hybrid lattice. The calculated void volume corresponds to 8.22% of the total unit-cell volume, indicating relatively compact packing. The residual voids are small, isolated and non-continuous, rather than forming extended cavities or interconnected channels. Their distribution reflects the geometrical constraints imposed by the rigid Bi–Br chains and planar organic cations, which limit perfect space filling in certain regions of the unit cell.

The limited void volume is consistent with the combined contributions of N–H⋯Br and N–H⋯O hydrogen bonding, π–π stacking interactions with an interplanar separation of 3.637 Å, and additional close intermolecular contacts. These interactions help organize the organic cations around the one-dimensional inorganic framework and consolidate the overall crystal packing. Consequently, the Hirshfeld surfaces, fingerprint plots and void analysis provide complementary evidence for the role of hydrogen bonding, aromatic stacking and other intermolecular contacts in stabilizing the structure of the title compound.

### 3.3. Vibrational studies

Complementary FT-IR and Raman spectroscopy were used to investigate the vibrational properties of the title compound, elucidating the nature of the organic cation, the integrity of the inorganic Bi–Br framework, and the intermolecular interactions that stabilize the hybrid lattice. The experimental spectra (Fig. 5 and 6), together with the calculated vibrational frequencies and assignments summarized in Table S3, provide a coherent and internally consistent description of the molecular and lattice dynamics.

**Fig. 5:**
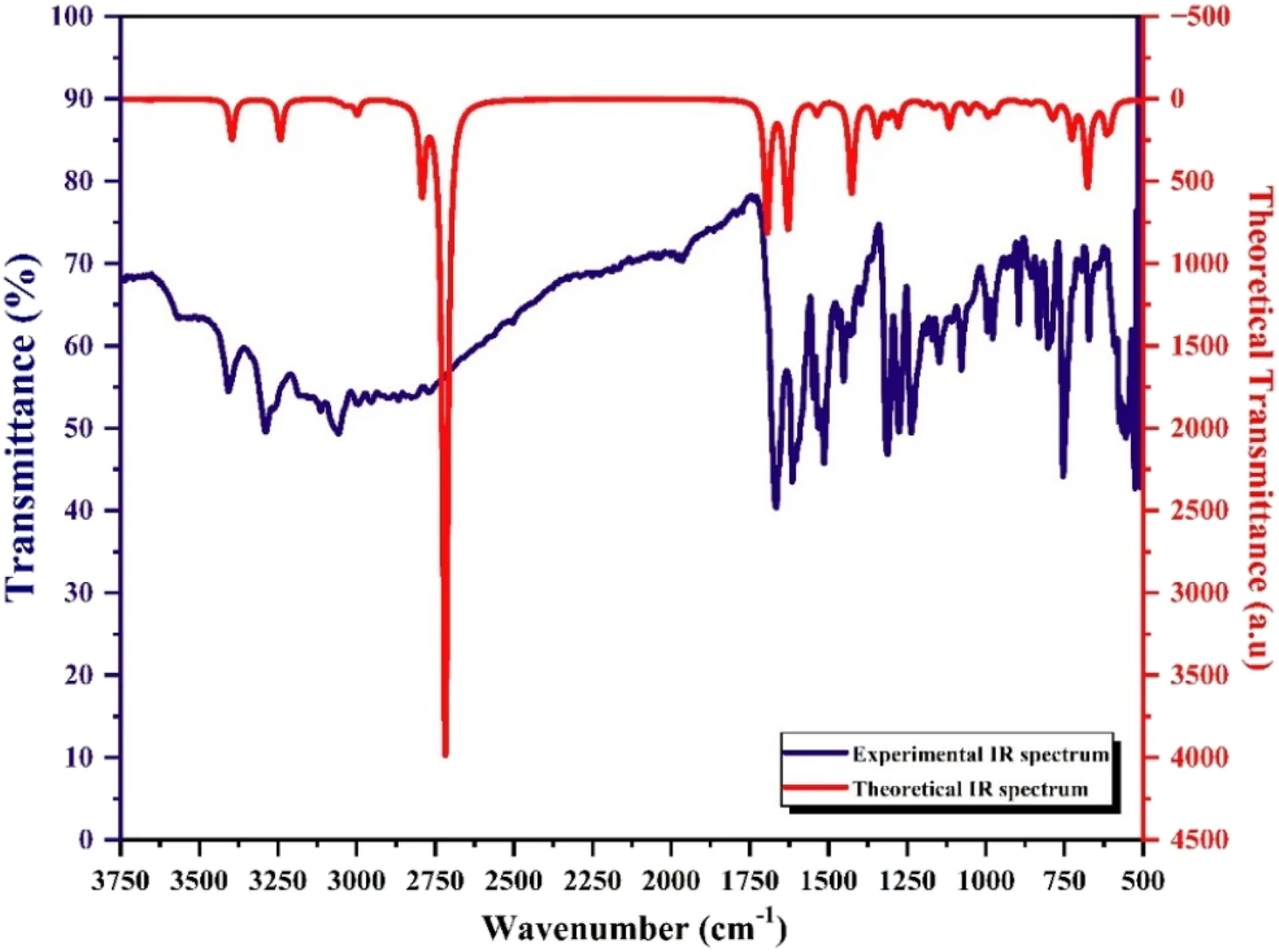
FT-IR spectrum of the title compound.

**Fig. 6:**
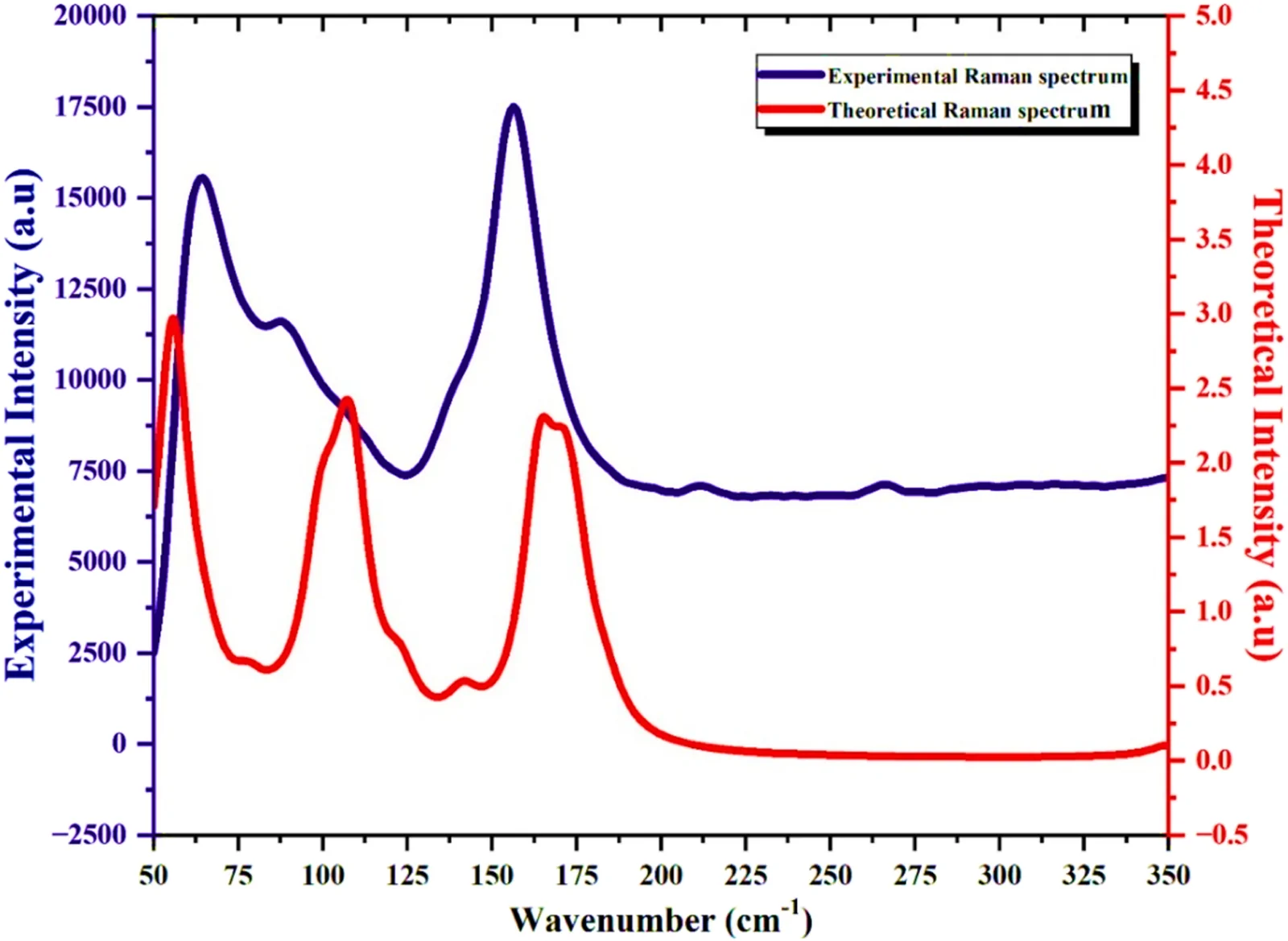
Raman spectrum of the title compound.

The theoretical FT-IR and Raman spectra were obtained from DFT harmonic frequency calculations on the optimized molecular structure and were used to assist in the assignment of vibrational modes. Note that the slight discrepancies observed in the 2500–3000 cm^−1^ region arise from the gas-phase approximation of the calculations, whereas the experimental spectra reflect solid-state hydrogen bonding and intermolecular interactions leading to band broadening.

In the high-frequency region of the FT-IR spectrum, the bands observed at 3406 and 3271 cm^−1^ are assigned to the asymmetric and symmetric stretching vibrations of the amide NH_2_ group, respectively. Their relatively high intensity and slight red shift with respect to isolated amide values reflect the involvement of these groups in intermolecular hydrogen bonding. The aromatic C–H stretching vibrations appear as weaker features at 3102 and 3048 cm^−1^, consistent with the heteroaromatic pyrazinamide skeleton. A distinct band at 2819 cm^−1^, together with a broad absorption envelope extending from 1950 to 2750 cm^−1^, is attributed to *ν*(N–H⋯Br) hydrogen-bonded stretching modes. The breadth and position of this feature provide direct spectroscopic evidence for strong N–H⋯Br interactions, in full agreement with the crystallographic hydrogen-bond analysis and Hirshfeld surface results.

The intense band centered at 1673 cm^−1^ corresponds unambiguously to the amide carbonyl *ν*(C=O) stretching vibration, confirming the preservation of the amide functionality within the hybrid framework and ruling out any transformation to carboxylate or other derivatives. In the mid-frequency region, the vibrational pattern is dominated by coupled modes involving the heteroaromatic ring and the amide linkage. The bands at 1608 and 1518 cm^−1^ are assigned to *ν*(C=N) and *ν*(C=C) stretching vibrations of the aromatic ring, respectively, reflecting the conjugated nature of the organic cation. The feature at 1444 cm^−1^ is mainly associated with NH_2_ bending, while the bands at 1315 and 1276 cm^−1^ arise from amide *ν*(C–N) stretching modes, indicative of strong electronic coupling between the amide group and the aromatic ring.

Further downfield, the band at 1247 cm^−1^ is attributed to in-plane C–H deformation, while the features at 1155 and 1138 cm^−1^ correspond to *β*(C–H) bending and *ν*(C–C) skeletal vibrations of the aromatic framework. The band at 1069 cm^−1^ is associated with NH_2_ deformation, whereas the feature at 979 cm^−1^ arises from aromatic C–H bending modes. The lower-frequency IR bands at 733 and 672 cm^−1^ are assigned to NH_2_ wagging and mixed C=C–N ring deformations, respectively, while the band at 576 cm^−1^ corresponds to collective C=N–C skeletal vibrations. These modes are particularly sensitive to the crystal packing environment and confirm the constrained geometry of the organic cations imposed by hydrogen bonding and π–π stacking interactions.

The Raman spectrum provides direct insight into the inorganic sublattice and lattice dynamics. The prominent Raman band at 157 cm^−1^ is attributed to Bi–Br stretching vibrations, while the band at 88 cm^−1^ corresponds to Br–Bi–Br bending modes, unequivocally confirming the integrity of the one-dimensional Bi–Br inorganic framework. The low-frequency Raman feature observed at 64 cm^−1^ is assigned to a lattice mode, reflecting collective vibrations involving both organic and inorganic components of the hybrid structure.

### 3.4. Thermogravimetric analysis

The thermal stability and decomposition pathway of (C_5_H_6_N_3_O)_2_·[BiBr_5_] were investigated by thermogravimetric analysis under an inert N_2_ atmosphere (Fig. 7). The compound remains thermally stable up to approximately 180 °C, beyond which three well-defined mass-loss events are observed in the TGA curve, each clearly resolved in the corresponding DTG profile. The first decomposition step extends from 180 to 270 °C, with a pronounced DTG maximum centered at 225 °C, and accounts for an experimental mass loss of 37.17%, in excellent agreement with the theoretical value of 37.55%. This stage is attributed to the combined release of one equivalent of Br_2_ together with partial fragmentation of the organic cations, yielding nitrogen-rich heterocyclic residues formally corresponding to two (C_4_H_5_N_2_) units. The early onset of this process reflects the relative lability of terminal halide species and the intrinsic thermal weakness of the amide moiety within the protonated pyrazinamidinium framework, which is known to undergo scission and deamidation under moderate heating.

**Fig. 7:**
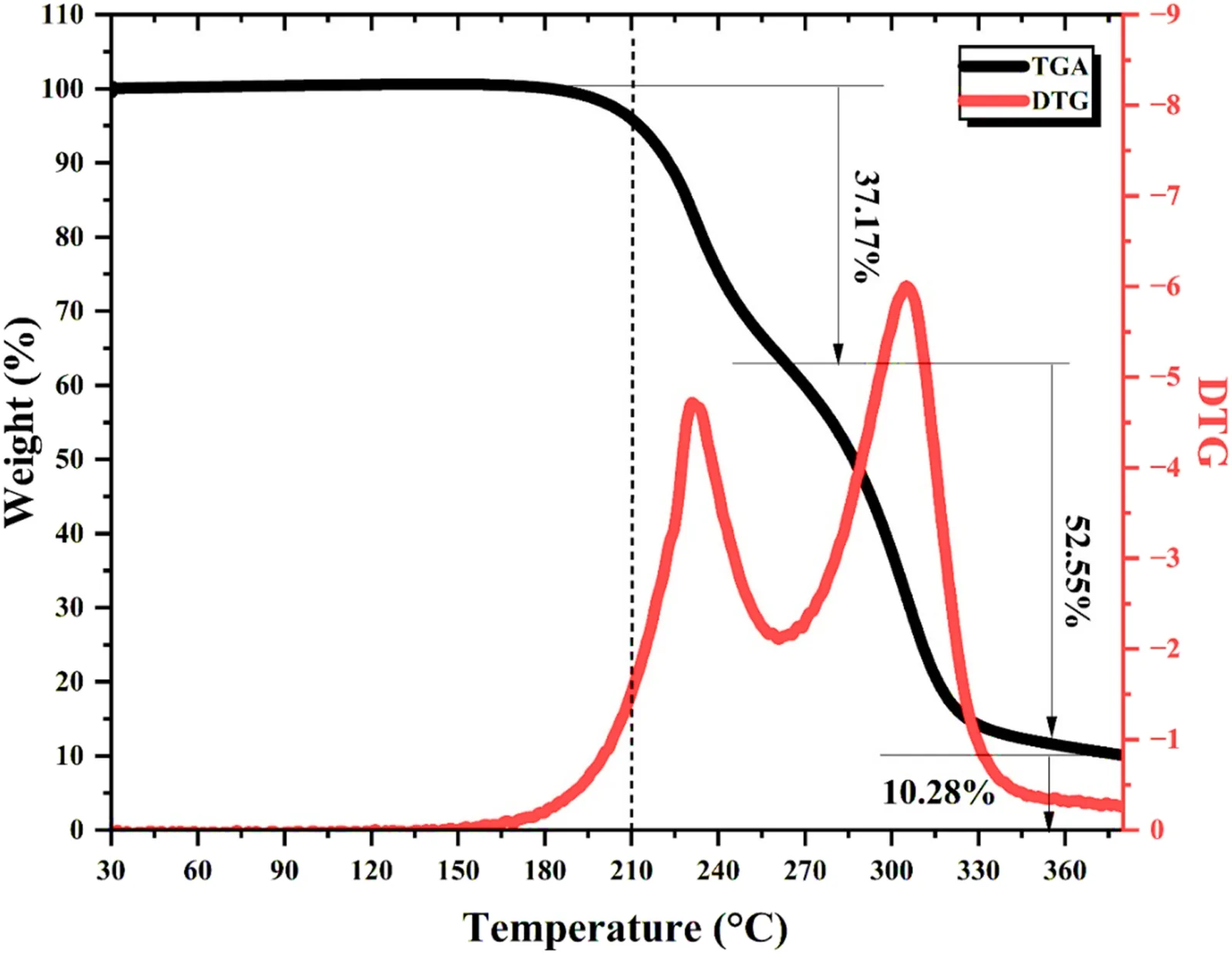
TGA thermal analysis curve of the title compound.

The second and most significant mass-loss event occurs between 270 and 360 °C, corresponding to an experimental weight loss of 52.55%, closely matching the calculated value of 52.42%. This step is unambiguously assigned to the complete volatilization of BiBr_3_, indicating full collapse of the inorganic backbone following removal of the organic stabilization. The excellent agreement between experimental and theoretical values, combined with the known volatility of BiBr_3_ at elevated temperatures, confirms that this stage represents the total elimination of the bismuth–bromide framework and marks the definitive destruction of the hybrid lattice.

Beyond 360 °C, the TGA curve reaches a plateau, leaving a final residue of 10.28%, which again matches remarkably well with the calculated value of 10.03%. Given that all bismuth and bromine species are fully accounted for in the preceding steps, this residual mass must necessarily correspond to a purely organic, nitrogen- and oxygen-rich condensed entity. At such elevated temperatures, small molecular fragments and aliphatic residues are thermodynamically unstable and cannot persist; therefore, the residue is most reasonably attributed to a highly condensed aromatic or heteroaromatic fragment originating from the thermal rearrangement of isocyanate-type species generated during the decomposition of the organic cations. While the precise molecular structure of this residual phase cannot be conclusively identified from TGA/DTG data alone, its mass, thermal robustness, and chemical constraints strongly support the formation of a compact, aromatized organic remnant, empirically consistent with a C_2_H_2_N_2_O_2_-type composition. The minor experimental deviation is attributed to instrumental interruption near the end of the run; nevertheless, the explored temperature range successfully captures the complete decomposition sequence of the hybrid material.

### 3.5. Optical study

#### 3.5.1. Diffuse reflectance spectroscopy (DRS)

Fig. 8 presents the UV-vis absorbance and reflectance spectra of (C_5_H_6_N_3_O)_2_·[BiBr_5_], showcasing four principal optical transitions whose energies and intensities reflect the composite electronic structure of the hybrid material.^42^ The high-energy peak at 245 nm arises from a π → π* transition within the pyrazinamidinium cation, corresponding to aromatic ring excitation involving conjugated π orbitals. The second absorption band at 275 nm is assigned to a high-energy electron–hole transition, situated above the HOMO–LUMO frontier level and representing excitation into higher unoccupied molecular states; this band reflects the intrinsic electronic structure of the organic subsystem and signifies participation of excited π* or Rydberg-like levels in the short-wavelength region. In contrast, the broad band at 375 nm corresponds to a distinct Ligand-to-Metal Charge Transfer (LMCT) process,^43,44^ where electron density is transferred from the heteroatom-rich ligand environment (primarily N and O lone pairs) toward the low-lying Bi(iii) acceptor orbitals. This LMCT behavior is consistent with the strong donor capability of the pyrazinamidinium functionality and the high polarizability of the Bi center. The most consequential transition occurs at 425 nm, corresponding to the intra-framework charge-transfer excitation that defines the fundamental optical response: this feature reflects electron promotion from the Br 4p-dominated valence band to the Bi 6p/organic π–mixed conduction band*, matching the HOMO → LUMO character predicted by DFT and PDOS analyses. This transition governs the onset of absorption in the visible region and is the electronic precursor to the measured bandgap.

**Fig. 8:**
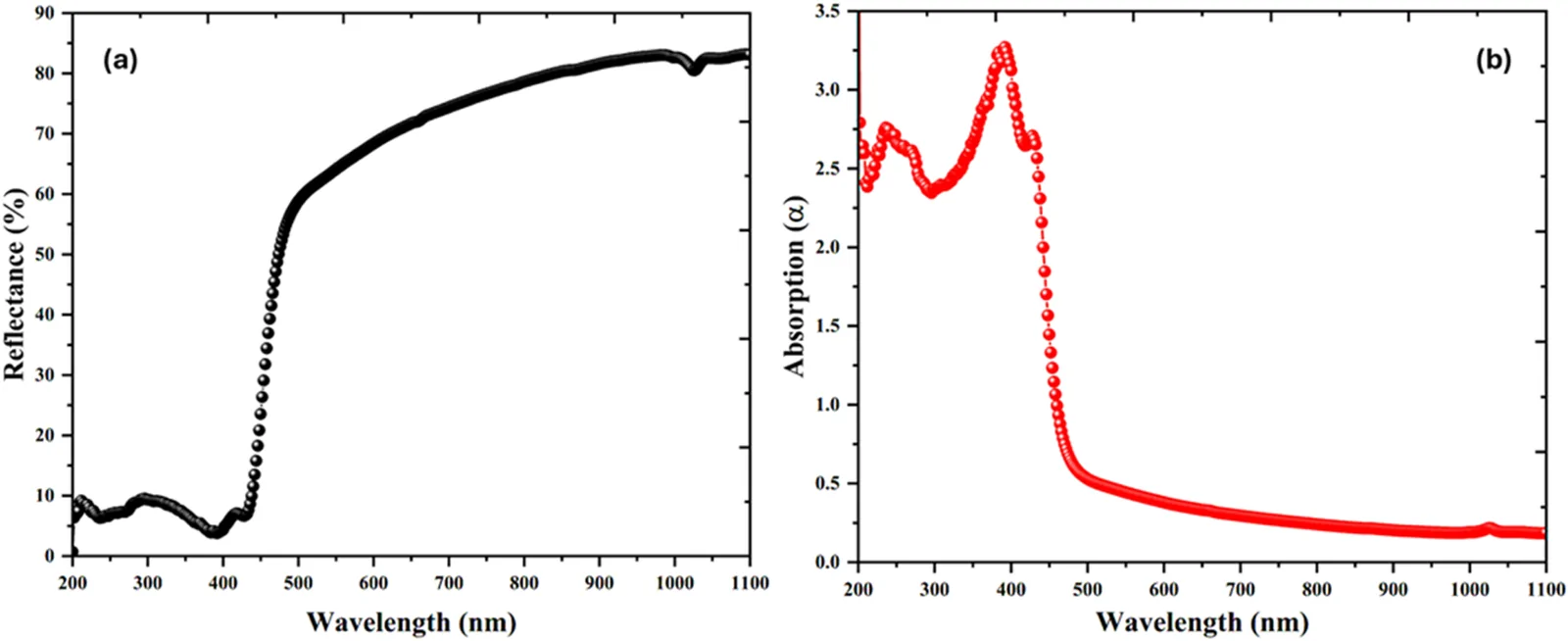
(a) Diffuse reflectance and (b) absorption spectrum.

The absorption edge appears sharply at ≈450 nm, with a precisely extracted threshold wavelength of 456 nm, characteristic of a direct forbidden-allowed transition typical of Bi-based halide frameworks exhibiting strong spin–orbit coupling. This sharp edge, with no extended sub-gap tailing, is accompanied by a single, dominant derivative peak, confirming that only one principal electronic transition governs the band-edge behavior, which is a signature of direct-gap semiconductors. Reflectance data further support this conclusion: the abrupt reflectance drop and mirrored increase in absorbance in the edge region signal a strong direct transition rather than an indirect phonon-assisted process. Additionally, the Urbach energy, determined to be 0.175 eV (∼6.25% of *E*_g_), is exceptionally low for hybrid halometallic materials, indicating minimal electronic disorder, low defect density, and a highly ordered ligand field surrounding the BiBr_6_ chains. Such a low Urbach energy again reinforces the direct-gap nature and demonstrates that the crystalline lattice preserves sharp electronic band edges with negligible tail states.

Collectively, the four assigned transitions, which are aromatic π → π* excitation (245 nm), high-energy electron–hole transition (275 nm), LMCT band (375 nm), and the crucial intra-framework CT transition at 425 nm, together with the sharp absorption edge and low Urbach energy, conclusively establish (C_5_H_6_N_3_O)_2_·[BiBr_5_] as a direct 2.8 eV semiconductor. The spectral features reflect the cooperative interaction between organic and inorganic components and form a coherent foundation for the subsequent bandgap analysis and optical-constant calculations presented in the next figures.

Fig. 9 shows the bandgap determination of (C_5_H_6_N_3_O)_2_·[BiBr_5_] using the Kubelka–Munk (KM) transformed reflectance data, where the optical function *F*(*R*) is employed to emulate the absorption coefficient for powdered and microcrystalline materials. The Kubelka–Munk function, defined as:^45,46^
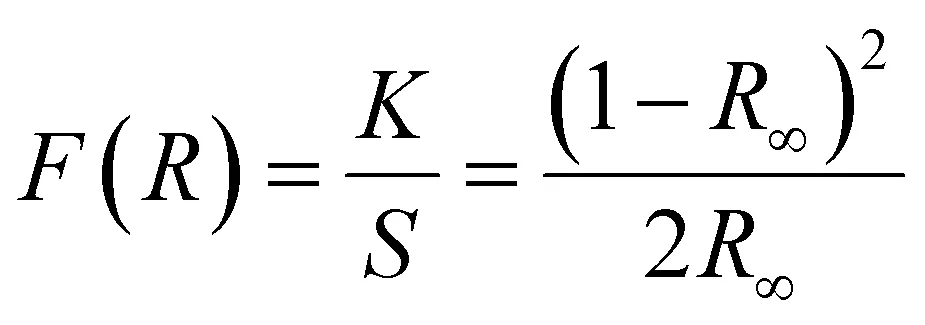


**Fig. 9:**
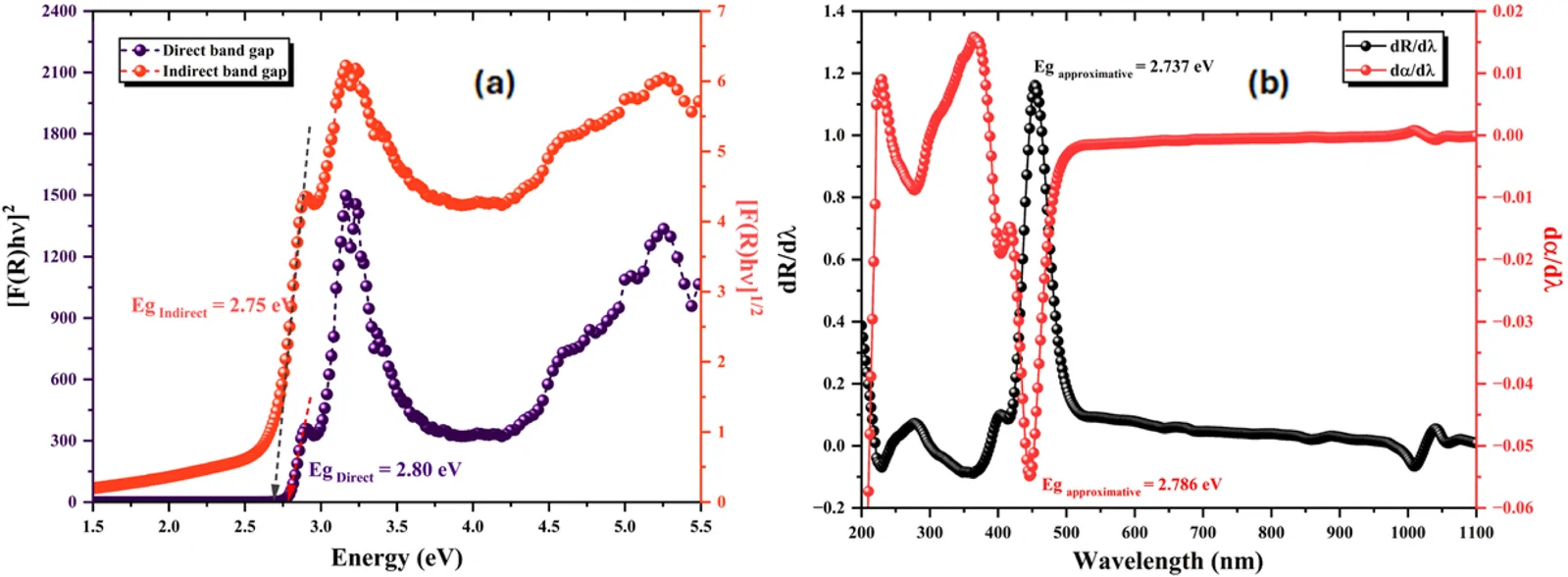
(a) Direct *vs.* indirect band gap determination *via* Tauc plot, (b) derivative plots of absorption and reflectance spectra.

In this context, *R* represents the absolute reflectance of the sample, measured using an integrating sphere system. *K* is the light absorption coefficient, and *S* is the scattering coefficient. The function *F*(*R*) is known as the Kubelka–Munk function. According to inter-band absorption theory, the absorption coefficient near the threshold, in relation to incident energy, can be described by the following equation:^47,48^
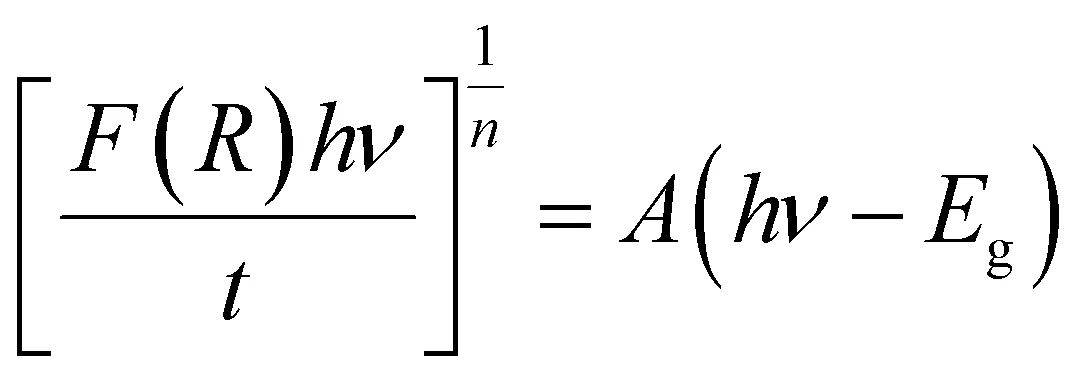


The Tauc formalism is applied using [*F*(*R*)*hν*]^2^ plotted against photon energy, a method specifically appropriate for evaluating direct allowed electronic transitions. The resulting Tauc plot exhibits a single, perfectly linear region near the band edge, with no curvature, multi-slope behavior, or secondary inflection points. This strict linearity is the defining criterion of a direct bandgap semiconductor, reflecting that the electronic transition responsible for the band edge obeys the direct allowed selection rules without requiring phonon participation. Extrapolation of the linear segment to the energy axis yields an experimental bandgap of 2.8 eV, consistent with the sharp absorption threshold previously observed in the UV-vis analyses.

The Tauc linearity is not only steep but also exceptionally coherent, reflecting a clean and unperturbed edge region devoid of mid-gap states or structural disorder that would distort the KM-based optical response. The absence of any pre-edge curvature or extended tailing demonstrates that the intrinsic transition from the valence band to the conduction band dominates the optical behavior, a feature typical of hybrid Bi-based semiconductors with strong internal ordering and well-defined inorganic–organic electronic coupling. Moreover, the linear region extends over a comparatively large energy window, indicating that the density of states near the band edges rises abruptly, again characteristic of direct-gap materials. This behavior mirrors the nature of the electronic structure revealed by the DOS and PDOS calculations, where the valence band is composed mainly of Br 4p states, and the conduction band minimum includes contributions from Bi 6p orbitals with additional mixing from the π* manifold of the organic cation.

The derivative analysis further supports this conclusion: the first derivative of the Kubelka–Munk function exhibits a single, intense, and sharply defined extremum, corresponding to a dominant electronic transition at the band edge. Direct semiconductors typically exhibit this behavior because the momentum of the electron and hole does not change during excitation, resulting in an abrupt change in optical response detectable through derivative spectra. The absence of multiple derivative peaks eliminates the possibility of overlapping transitions or indirect-gap processes, leaving the direct-gap interpretation unequivocal.

The small difference between the experimental bandgap (2.8 eV) and the DFT-calculated HOMO–LUMO gap (∼2.5 eV) is entirely consistent with the limitations of Gaussian-based calculations for extended inorganic–organic hybrid materials. Gaussian, operating within molecular or cluster-based approximations, cannot fully simulate the periodicity and dimensionality of one-dimensional chains or higher-symmetry extended solids. As a result, DFT calculations performed using finite molecular fragments systematically underestimate the bandgap, because they do not reproduce the long-range periodic potential, bandwidth dispersion, or excitonic effects that stabilize the true crystalline electronic structure. The excellent semi-quantitative agreement (0.3 eV deviation) therefore reflects not an inconsistency but the expected behavior of hybrid systems when comparing periodic experimental properties to non-periodic DFT cluster models.

Fig. 10(a) quantifies the Urbach energy (*E*_U_) of (C_5_H_6_N_3_O)_2_·[BiBr_5_], offering a sensitive probe of the degree of structural and electronic disorder near the optical band edge. The formula used is:^49^
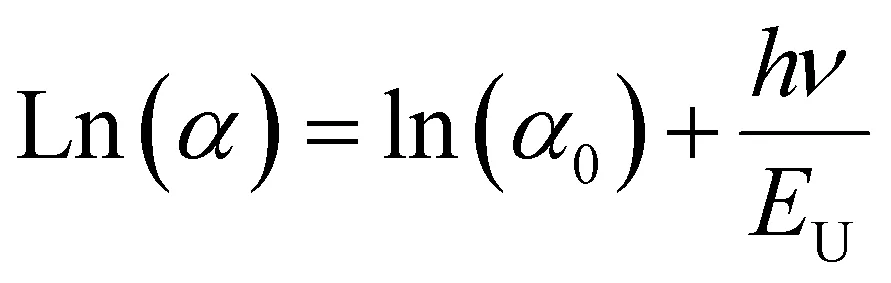


**Fig. 10:**
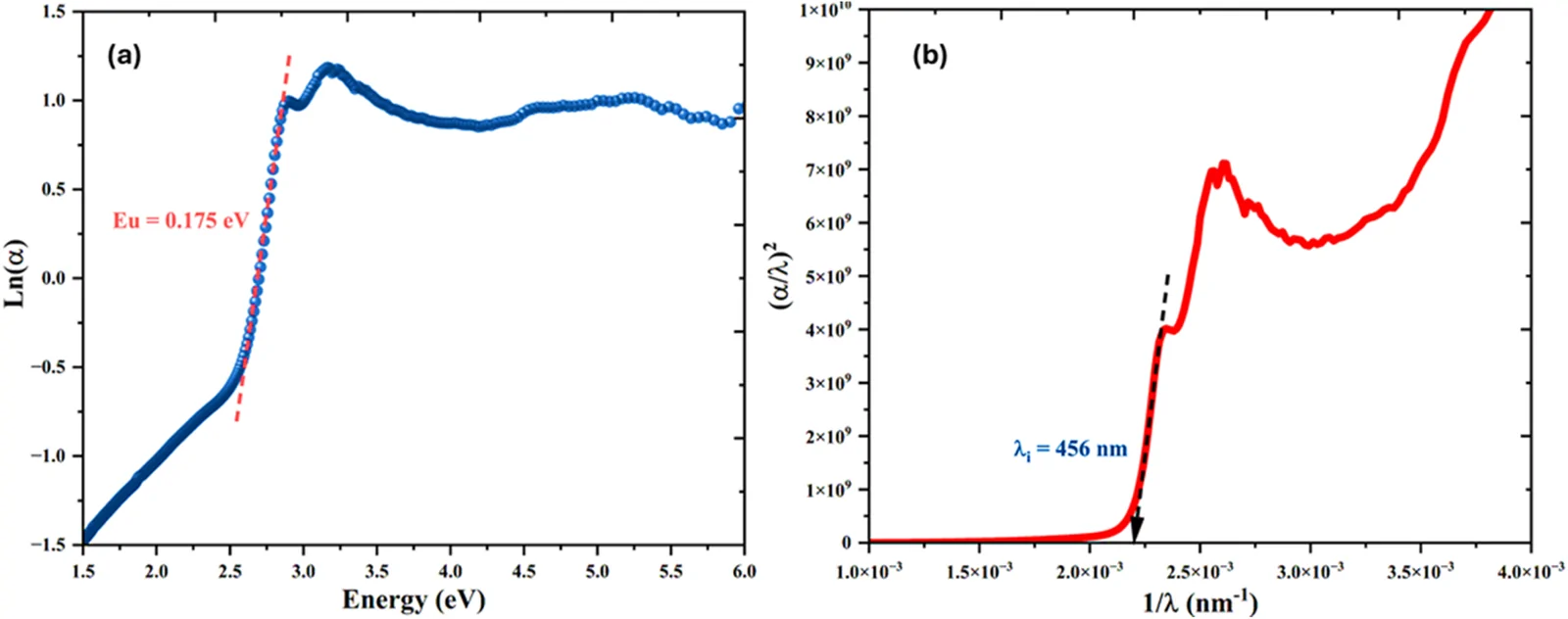
(a) Urbach energy analysis, (b) variation of the (*α*/*λ*)^2^ curve as a function of 1/*λ*.

The Urbach plot, constructed from the exponential region of the Kubelka–Munk tail, yields a remarkably low value of *E*_U_ = 0.175 eV. Such a small Urbach energy is a clear indicator of minimal structural fluctuations, low phonon-assisted broadening, and negligible defect-induced tailing of the electronic states. For hybrid materials, especially those containing heavy halides, typical values often exceed 0.25–0.40 eV due to lattice softness; therefore, an Urbach energy of 0.175 eV situates this compound among the most optically ordered Bi-based hybrids, reflecting the substantial rigidity imparted by the BiBr_6_ octahedron, the stabilizing hydrogen-bonding network, and the pseudo-2D halogen packing identified previously.

The low *E*_U_ value also confirms the direct nature of the bandgap identified through both threshold wavelength and Kubelka–Munk Tauc analysis. Direct semiconductors characteristically exhibit very steep absorption edges with small Urbach energies because the electronic transition occurs without phonon involvement; the density of states increases sharply at the band edge, leaving little room for thermal or disorder-related smearing. In this material, the measured *E*_U_ represents only 6.25% of the experimental bandgap, a ratio that is exceptionally small and consistent with high-quality crystalline order. The absence of any significant exponential tail preceding the absorption edge implies that the optical transition is dominated entirely by the intrinsic charge-transfer excitation, without interference from sub-gap defect transitions or structural distortions. This behavior supports the interpretation that the BiBr_6_ chain is electronically homogeneous and that the organic cations do not introduce local electronic fluctuations that would broaden the absorption tail.

Fig. 10(b) presents the determination of the threshold wavelength (*λ*_i_) for (C_5_H_6_N_3_O)_2_·[BiBr_5_], providing a direct evaluation of the optical absorption onset and the steepness of the band edge:^50,51^
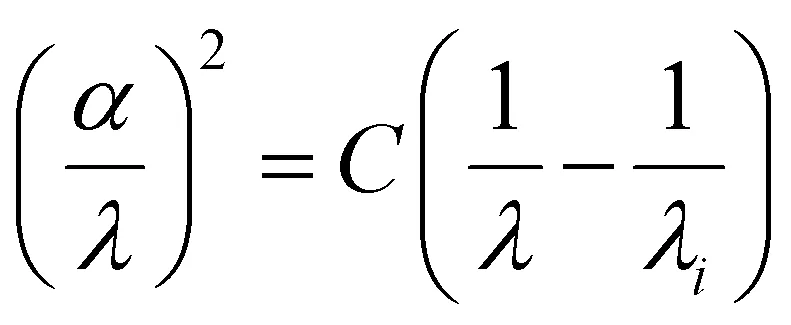


The spectrum shows a clean, abrupt, and well-defined transition between the transparency region and the onset of strong absorption, allowing the threshold wavelength to be precisely extracted at 456 nm. This sharp cutoff is characteristic of a direct allowed electronic transition, reflecting an immediate and strong increase in optical absorption once the photon energy surpasses the intrinsic bandgap value. The absence of any shoulder, slope discontinuity, or pre-edge curvature indicates that the material possesses minimal tail-state contributions and a low density of localized defect states, which is uncommon in hybrid halide systems unless the crystalline lattice is highly ordered and the octahedral units are structurally coherent. The threshold wavelength of 456 nm corresponds directly to an optical bandgap of 2.8 eV, in perfect agreement with the Tauc analysis derived from the Kubelka–Munk transform. This convergence of results affirms that the absorption onset is a fundamental transition from the Br 4p-dominated valence band to the Bi 6p/π* hybridized conduction band, consistent with the PDOS interpretation.

The sharpness of the band edge reflects the intrinsic structural features of the compound: the linear Bi–Br connectivity, the well-defined one-dimensional chains, and the highly directional hydrogen-bonding and halogen-driven supramolecular stabilization collectively suppress lattice disorder, resulting in negligible absorption tailing below the onset. The threshold wavelength also shows that no extrinsic absorption centers, such as surface defects, impurities, or localized trap states, intervene before the onset of the fundamental transition, highlighting the optical purity of the crystal. This sharp optical boundary is a critical identifier of a direct-gap semiconductor and defines the optical activity region for the subsequent analysis of refractive index, extinction coefficient, and conductivity in later figures.

The penetration depth (*δ*), also known as skin depth, indicates how far incident light travels before its intensity drops to approximately 37% (1/*e*) of the original. This measure offers valuable insights into surface and bulk properties. It varies based on the material's absorption and scattering characteristics: highly transparent materials allow deeper light penetration, whereas highly absorbent materials result in shallower penetration. The formula for penetration depth is provided by:^52^
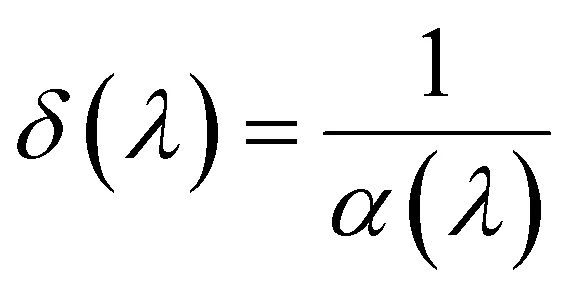


Fig. 11 illustrates the wavelength-dependent skin depth (*δ*) of (C_5_H_6_N_3_O)_2_·[BiBr_5_], revealing three distinct optical penetration regimes that directly reflect the material's electronic structure, absorption behavior, and band-edge position. In the 200–450 nm interval, *δ* remains essentially constant at ∼0.40 cm, indicating extremely strong absorption in the high-energy UV region where the electronic transitions (π → π*, LMCT, and higher-energy charge-transfer bands) dominate. The constancy of *δ* in this region shows that the absorption coefficient (*via* the Kubelka–Munk formalism) is sufficiently high that photons penetrate only a shallow depth before being fully attenuated. This behavior is characteristic of direct semiconductors with intense interband transitions lying well above the bandgap energy, confirming that the lattice responds uniformly to incoming high-energy photons without the formation of pre-edge states or variation in the local density of states.

**Fig. 11:**
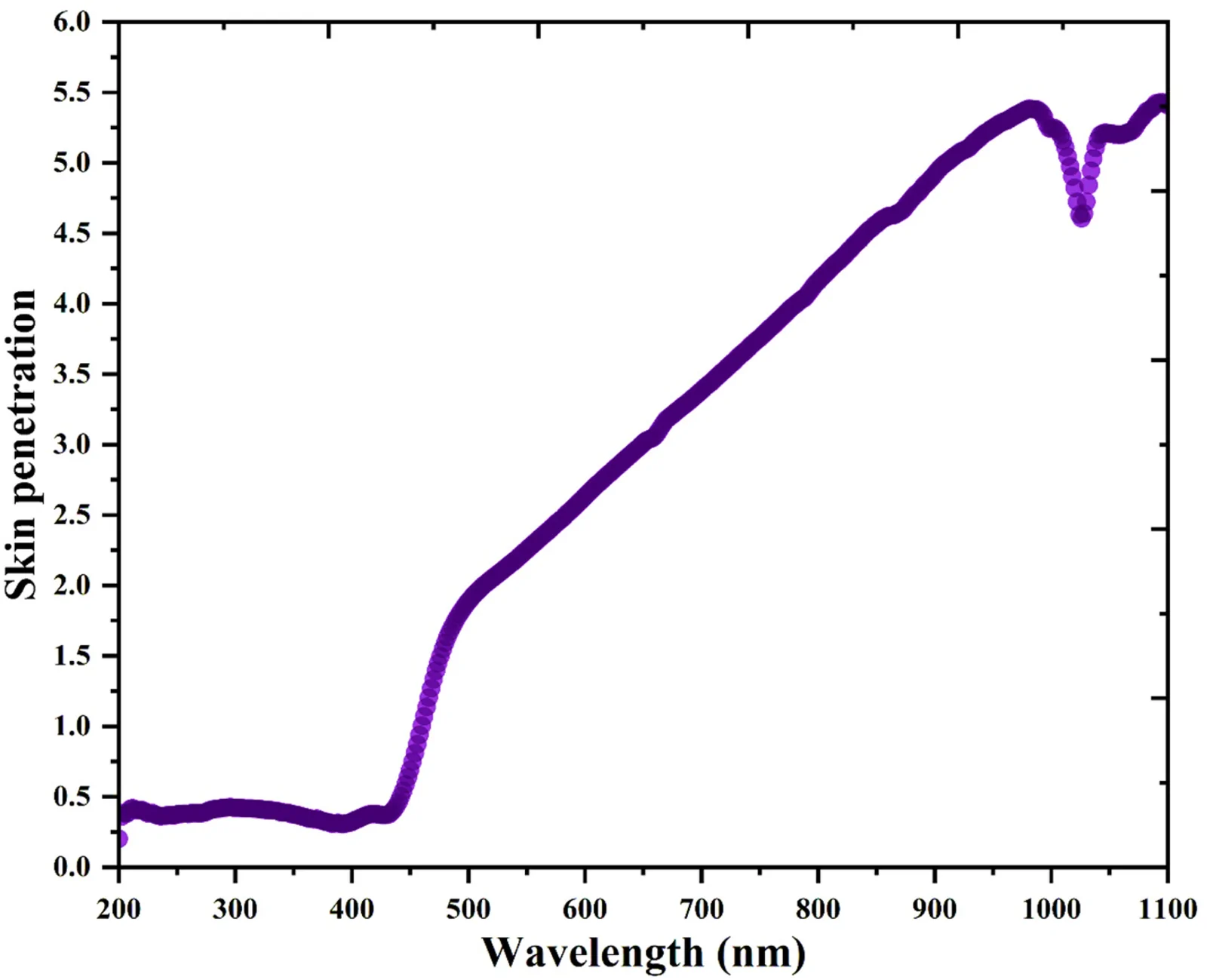
Variation of penetration depth (*δ*) with wavelength (*λ*).

At wavelengths above the absorption edge (∼450 nm), the material transitions into a second optical regime: between 450 and 950 nm, the skin depth increases continuously from 0.40 cm up to about 5.5 cm. This steady rise reflects the dramatic drop in the absorption coefficient once the photon energy falls below the 2.8 eV bandgap, causing photons to penetrate increasingly deeper into the lattice. The broadness and smoothness of this intermediate region indicate that the compound has minimal sub-gap defect absorption, consistent with the low Urbach energy (0.175 eV), and thus the attenuation is governed purely by intrinsic electronic transitions rather than disorder-induced tail states. The monotonic increase of *δ* throughout this region is also typical of crystalline hybrid halometalates whose valence-band density decreases abruptly below the direct transition threshold, allowing near-visible and near-infrared photons to travel significant distances without substantial interaction with electronic states.

Finally, the third regime emerges above 950 nm, where the skin depth reaches a plateau at ∼5.5 cm, indicating that the absorption coefficient becomes extremely small, effectively rendering the material transparent to near-infrared radiation. This optical transparency window signifies that no active electronic, excitonic, or vibrational overtones fall within this energy range. The constant *δ* in this region is associated with the absence of low-energy intraband transitions, consistent with the wide separation between conduction-band minima and any potential defect states. The deep NIR penetration further confirms the electronic cleanliness of the system and reveals its potential for applications where controlled partial transparency is required, such as photonic waveguides, optical windows, and NIR sensing modules, while maintaining strong absorption in the UV-visible region.

The optical extinction coefficient *k*(*λ*) describes the combined effect of absorption and scattering of light within the material, providing direct insight into its electronic transitions, and is expressed as:^53^
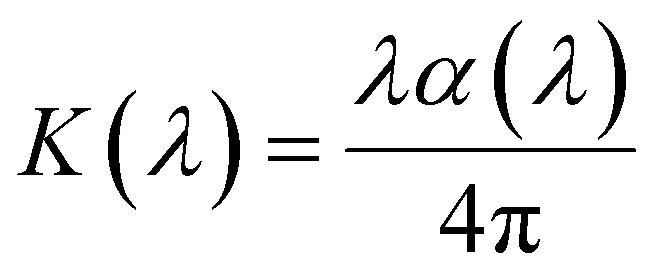


The refractive index (*n*) quantifies the interaction between incident light and the material's electronic structure, reflecting its polarizability and optical density. It was computed using the following relation:^54^
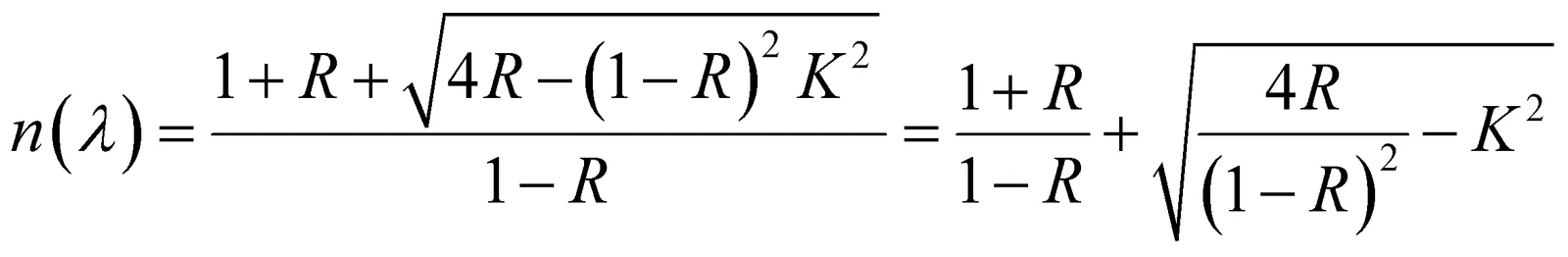


Fig. 12 presents the dispersion profiles of the refractive index (*n*) and the extinction coefficient (*k*) for (C_5_H_6_N_3_O)_2_·[BiBr_5_], providing a detailed view of how the material interacts with electromagnetic radiation across the UV-vis-NIR range and allowing direct correlation of optical constants with the underlying band structure. The refractive index curve exhibits a pronounced dispersion in the UV region (200–300 nm), where *n* reaches its maximum. This high-index region corresponds to the spectral domain where strong interband transitions occur (π → π*, LMCT, and high-energy CT bands). The rapid variation of *n* in this region is typical of direct semiconductors, where a large density of electronic states in proximity to the high-energy absorption bands induces a strong wavelength dependence through the Kramers–Kronig relation. As the wavelength increases toward the absorption edge (∼450 nm), the dispersion of *n* decreases gradually, and once past the bandgap region (visible to NIR), *n* stabilizes into a smooth, asymptotic profile, reflecting the absence of additional electronic transitions and the flattening of the dielectric response.^55–58^

**Fig. 12:**
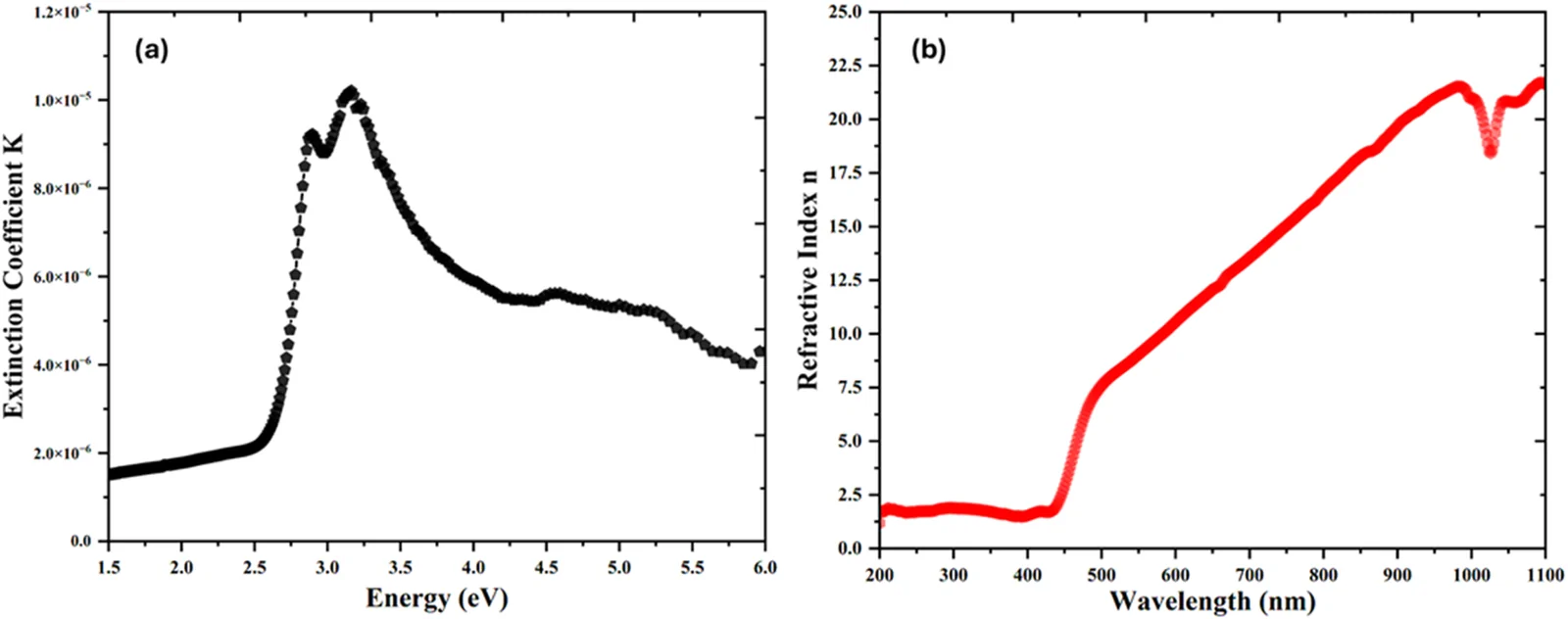
(a) Variation of extinction coefficient (*K*) as a function of energy, (b) refractive index (*n*) plot.

The extinction coefficient *k*, which directly tracks the material's absorption coefficient *via* the Kubelka–Munk formalism, displays a complementary behavior. In the far-UV region (200–300 nm), *k* exhibits strong and sharply defined peaks, corresponding to the intense high-energy transitions identified in the absorption spectrum. As the wavelength approaches the bandgap threshold, *k* decreases steeply, mirroring the sharp absorption edge characteristic of a direct bandgap semiconductor. The nearly vertical drop of *k* at ∼450 nm highlights the extremely abrupt transition between absorbing and transparent regimes, consistent with the direct nature of the 2.8 eV bandgap and the low Urbach energy (0.175 eV). Beyond ∼500 nm, *k* becomes very small, confirming the material's high transparency in the visible-NIR range and explaining the large skin depth and low absorption coefficients previously observed.

The interplay between *n* and *k* across the energy spectrum provides a deeper interpretation of the photophysical behavior of the material. The strong dispersion of *n* near the band edge indicates substantial photon–electron coupling, which is typical of lead-free halometalates where Bi-based octahedra dominate the optical response through heavy-atom p-orbital participation. This behavior is also consistent with the PDOS, which shows that the upper valence band is primarily Br-4p in character, while the conduction band has significant Bi-6p contributions; such orbital distributions produce strong dipole-allowed transitions that directly influence both *n* and *k*. The suppression of *k* in the visible and NIR regions, accompanied by the flattening of *n*, confirms that no mid-gap electronic states or low-energy charge-transfer processes interfere with the intrinsic band structure. This reinforces the material's optical purity and enhances its suitability for applications requiring controlled light propagation, such as dielectric layers, UV-vis filters, and optoelectronic components.

The optical conductivity (*σ*_op_) describes the response of the material's charge carriers under an oscillating electromagnetic field, bridging its optical and electronic properties. It was calculated using the relation:
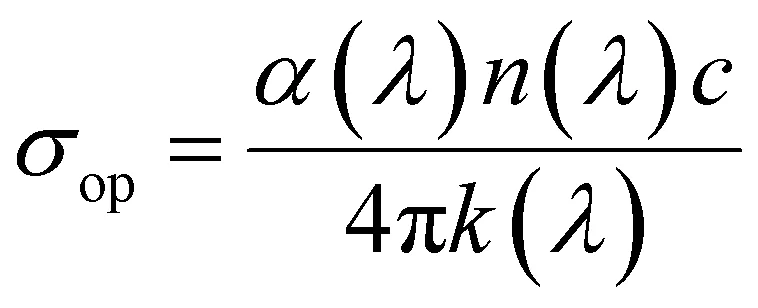


Fig. 13 presents the wavelength-dependent optical conductivity (*σ*_op_) of (C_5_H_6_N_3_O)_2_·[BiBr_5_], providing a direct probe of the material's ability to generate and mobilize photoinduced charge carriers under electromagnetic excitation. The profile of *σ*_op_ mirrors the electronic landscape revealed by the absorption, *k*, and Kubelka–Munk analyses, but it also introduces a deeper picture of how efficiently the electronic structure couples to incoming photons across the full UV-vis-NIR spectrum. In the 200–350 nm region, *σ*_op_ reaches its highest values, forming a broad and intense conductivity plateau associated with the strong π → π*, LMCT, and high-energy CT transitions originating from the Br-4p-dominated valence states toward the Bi-6p and π* components of the conduction band. These transitions involve orbitals with high spatial overlap and strong dipole activity, leading to efficient photon absorption and immediate formation of highly mobile excited-state carriers. This high-conductivity plateau is a hallmark of direct-gap systems with dense electronic states at high energies and confirms the robust charge-transfer capability intrinsic to the BiBr_6_ framework.

**Fig. 13:**
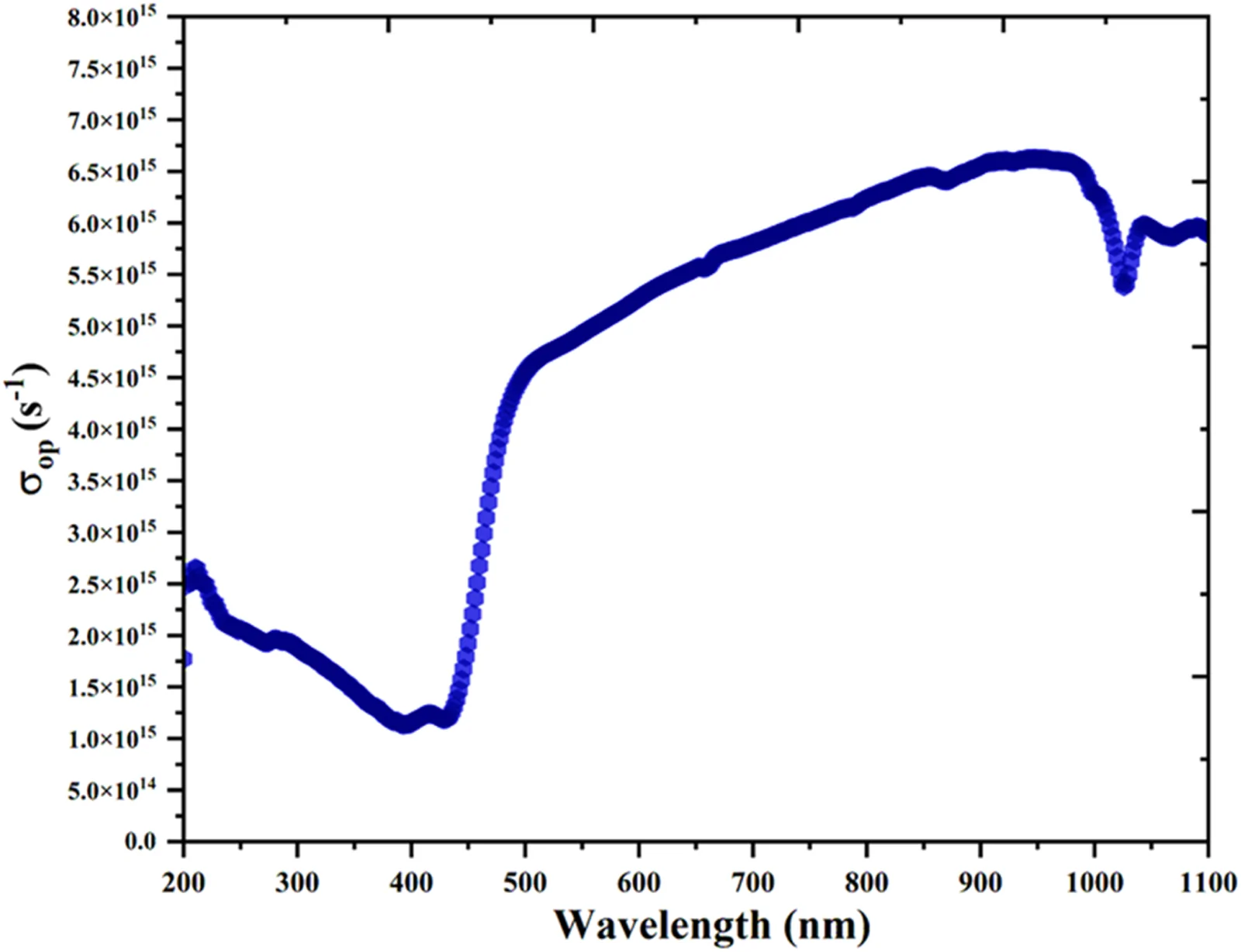
Optical conductivity plot.

At wavelengths approaching the bandgap (around 450 nm, 2.8 eV), *σ*_op_ exhibits a sharp decline, faithfully tracking the abrupt absorption edge and the steep collapse of the extinction coefficient. This nearly vertical fall is a diagnostic signature of a direct allowed transition, where the joint density of states drops instantaneously once photon energy falls below the bandgap threshold. The spectral discontinuity in *σ*_op_ indicates minimal disorder-mediated pathways, consistent with the extremely low Urbach energy (0.175 eV), and confirms the absence of sub-gap excitations or defect-induced conductivity channels. Unlike indirect semiconductors, which typically show a gradual tailing of *σ*_op_ due to phonon-coupled transitions, the crystal here exhibits a band-edge response that is clean, self-consistent, and structurally coherent.

In the visible to NIR region (*λ* > 500 nm), *σ*_op_ becomes very low and nearly featureless, reflecting the transparency of the material in this spectral window and the absence of low-energy charge-transfer processes or intraband excitations. The conductivity stabilizes at a minimal background value, indicating that any remaining interactions between photons and the electronic lattice are negligible. This plateau further confirms that the BiBr_6_ octahedral chains form an electronically uniform backbone without localized trap states or mid-gap impurity levels, ensuring that the optical response is governed exclusively by intrinsic interband transitions. The DOS and PDOS support this behavior: the conduction-band minimum is dominated by Bi-6p states with no low-energy satellite states, while the valence band decreases rapidly in density below the Br-4p edge, leaving no available transitions in the NIR regime.

Fig. 14 provides the total and partial density of states (DOS/PDOS) of (C_5_H_6_N_3_O)_2_·[BiBr_5_], allowing precise identification of the orbitals responsible for the electronic transitions observed experimentally. The DOS plot reveals a clean, well-defined bandgap of ∼2.5 eV at the DFT level, only slightly lower than the experimental optical gap of 2.8 eV. This small difference is fully expected: Gaussian-based DFT systematically underestimates the bandgap of one-dimensional and higher-dimensional hybrid halometalates, primarily because conventional functionals are not optimized to reproduce long-range electron correlation and dispersion effects present in extended inorganic substructures like the BiBr_6_ chains. Despite this intrinsic limitation, the theoretical and experimental bandgaps converge closely, confirming the reliability and internal consistency of the optical interpretation.

**Fig. 14:**
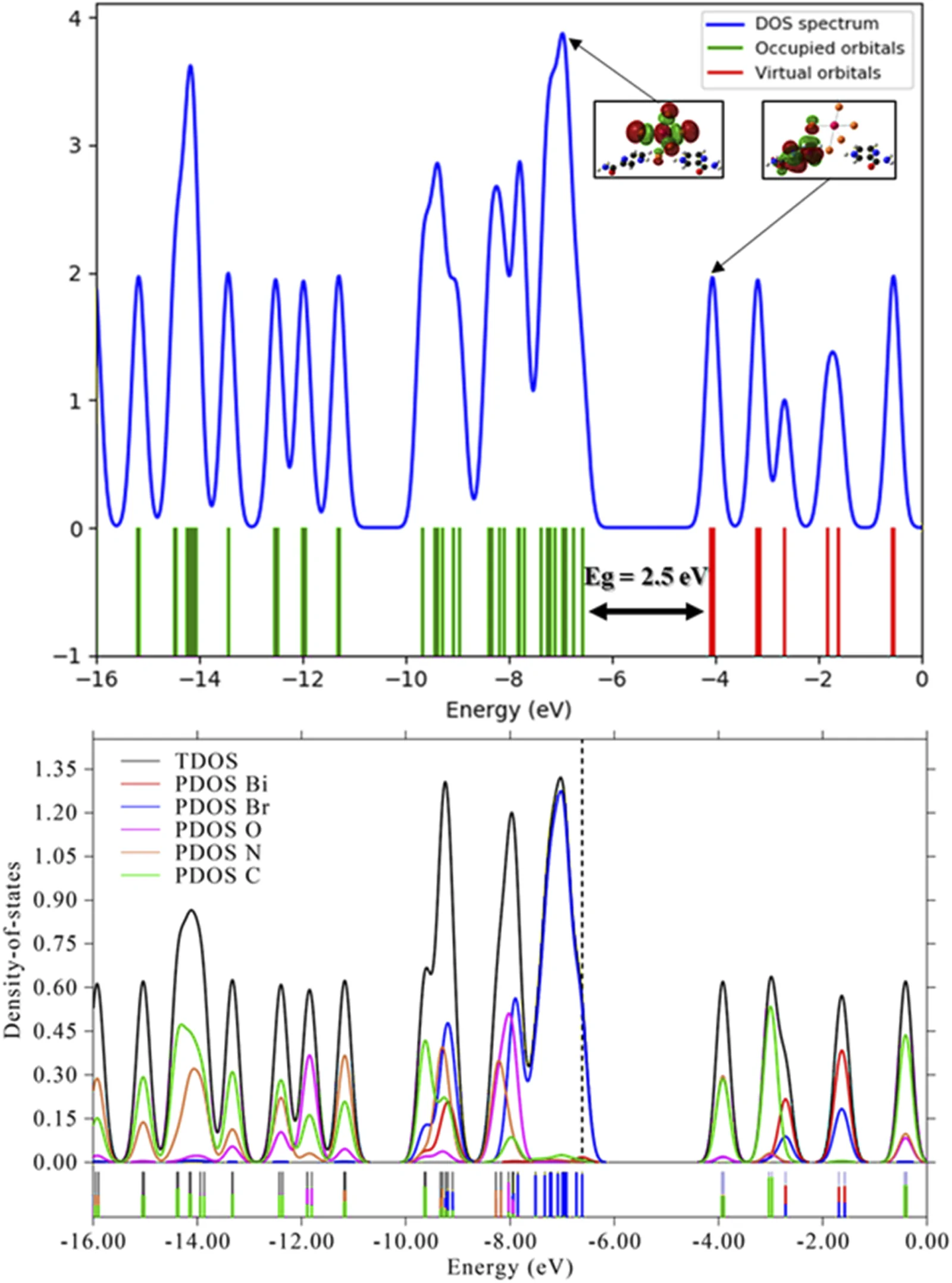
Total and partial densities of states (TDOS and PDOS).

The PDOS reveals that the upper valence band (VB) is overwhelmingly dominated by Br-4p orbitals, with minor contributions from the π-system of the protonated pyrazinamidinium cations. These Br-4p states are highly dispersed and provide the main reservoir of electrons participating in the optical transitions. In contrast, the conduction band (CB) is principally composed of Bi-6p orbitals, which form the electronic backbone of the BiBr_6_ octahedral chains, accompanied by a secondary contribution from the organic π* orbitals. This VB → CB orbital distribution is typical of bismuth-based halometalates and creates a strong dipole-allowed transition pathway, directly explaining the sharp absorption edge and the high intensity of the interband excitations.

A crucial aspect revealed by the PDOS is the identification of the last experimental absorption peak at ∼420 nm, which corresponds to a specific HOMO → LUMO charge-transfer excitation that couples inorganic and organic electronic manifolds. Here, the HOMO is primarily Br-4p in character, while the LUMO contains a measurable contribution from the π* orbitals of the pyrazinamidinium cations. This peak, therefore, represents a hybrid inorganic-to-organic charge transfer (IOCT) process, a type of transition frequently associated with enhanced photophysical tunability in low-dimensional hybrid materials. The PDOS clearly shows the energy alignment necessary for this IOCT transition: the π* states of the organic cations sit just above the Bi-6p conduction band minimum, forming a secondary electron-accepting channel that becomes optically accessible at energies slightly below the main inorganic transition manifold.

The DOS further demonstrates the absence of electronic trap states or mid-gap impurity levels, consistent with the extremely low Urbach energy (0.175 eV), the sharp absorption edge, and the clean conductivity spectrum. The VB tail decays abruptly with no intermediate states, and the CB presents a smooth onset with no localized features, indicating a structurally coherent and electronically homogeneous one-dimensional framework. This electronic purity directly matches the long-range structural regularity of the BiBr_6_ chains and the stabilizing pseudo-2D halogen packing previously discussed.

#### 3.5.2. Photoluminescence properties

The photoluminescence response of (C_5_H_6_N_3_O)_2_·[BiBr_5_], presented in Fig. 15, exhibits a dominant, remarkably sharp, and intense emission band centered at approximately 540 nm, corresponding to an emissive energy significantly lower than the optical band gap of 2.8 eV determined from diffuse reflectance analysis. The substantial Stokes shift indicates pronounced excited-state structural relaxation prior to radiative recombination.

**Fig. 15:**
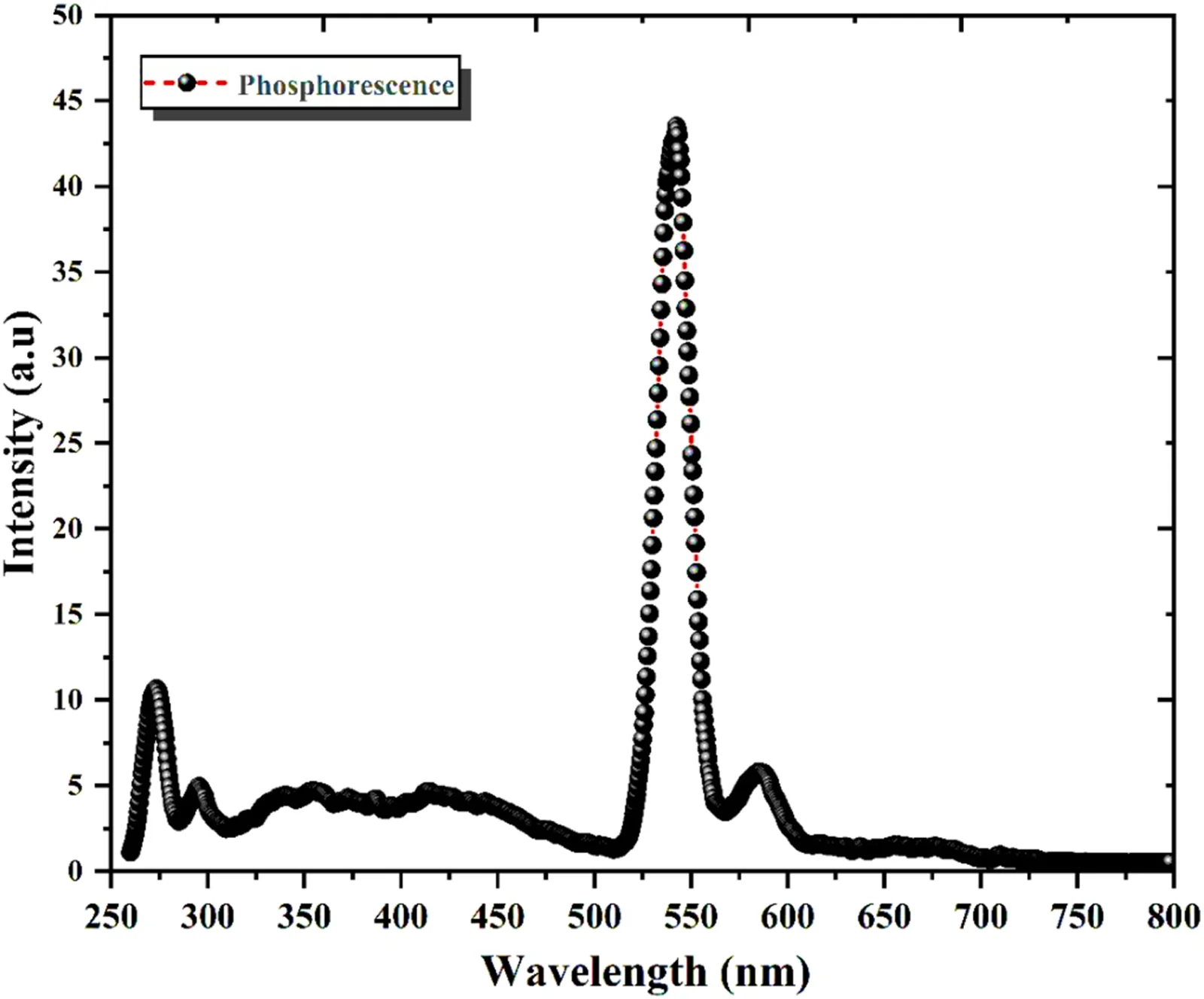
Photoluminescence emission plot.

The emission band displays moderate broadening, characteristic of localized excited states in low-dimensional Bi–Br hybrid frameworks, where electron–phonon coupling contributes to spectral width. Weak higher-energy shoulders are observed in the 250–500 nm region; however, their very low intensity confirms that they represent minor secondary radiative channels rather than dominant recombination pathways. Their presence does not indicate significant defect-related emission, supporting the overall optical quality of the crystalline phase.

Given that the emission energy lies below the band-edge transition and considering the strong spin–orbit coupling associated with Bi^3+^ (6s^2^ configuration), efficient intersystem crossing is expected in this heavy-atom-containing system. Such conditions are known to favor triplet-state radiative recombination in low-dimensional bismuth halide hybrids.

While time-resolved measurements would be required for definitive kinetic confirmation, the ensemble of spectroscopic indicators, such as namely the pronounced Stokes shift, the emission energy positioned significantly below the optical band gap, and the structural rigidity of the BiBr_6_ coordination environment, collectively supports the assignment of the emission to a relaxed triplet excited state. The magnitude of the Stokes shift reflects substantial lattice relaxation prior to radiative recombination, consistent with self-trapped exciton formation in low-dimensional Bi–Br frameworks. Such localization is further stabilized by the asymmetric distortion of the inorganic octahedron and the surrounding hydrogen-bonded organic network, which together favor excited-state trapping and structural reorganization.

Under these conditions, and considering the strong spin–orbit coupling associated with the 6s^2^ electronic configuration of Bi^3+^, efficient intersystem crossing from singlet to triplet manifolds is highly probable. The resulting spin-forbidden T_1_ → S_0_ transition provides a physically consistent explanation for the observed emission characteristics. Accordingly, the radiative pathway is best described as originating from triplet-state recombination, strongly indicative of phosphorescent behavior rather than purely singlet fluorescence, while acknowledging that lifetime measurements would provide final confirmation.

This photophysical behavior is quantitatively represented in the CIE 1976 chromaticity diagram shown in Fig. 16, where the emission plots within the yellowish-green region, in perfect agreement with the 540 nm PL maximum. The chromaticity coordinates obtained from the experimental emission curve confirm a high purity of the emitted color, with no spectral contamination from secondary bands. The position of this coordinate is particularly relevant for optoelectronic applications: yellow-green emissions occupy an important window for high-efficiency down-conversion layers in photovoltaic architectures, where re-emission in this range enhances spectral matching with silicon-based absorbers. The CIE point also aligns closely with the region typically associated with high quantum sensitivity in photodetector and display technologies, indicating potential utility in light-harvesting or solid-state lighting devices based on bismuth halide hybrids. The excellent overlap between the PL peak and its CIE chromaticity signature demonstrates that the emission originates from a single well-defined radiative process, not from overlapping emissive states.

**Fig. 16:**
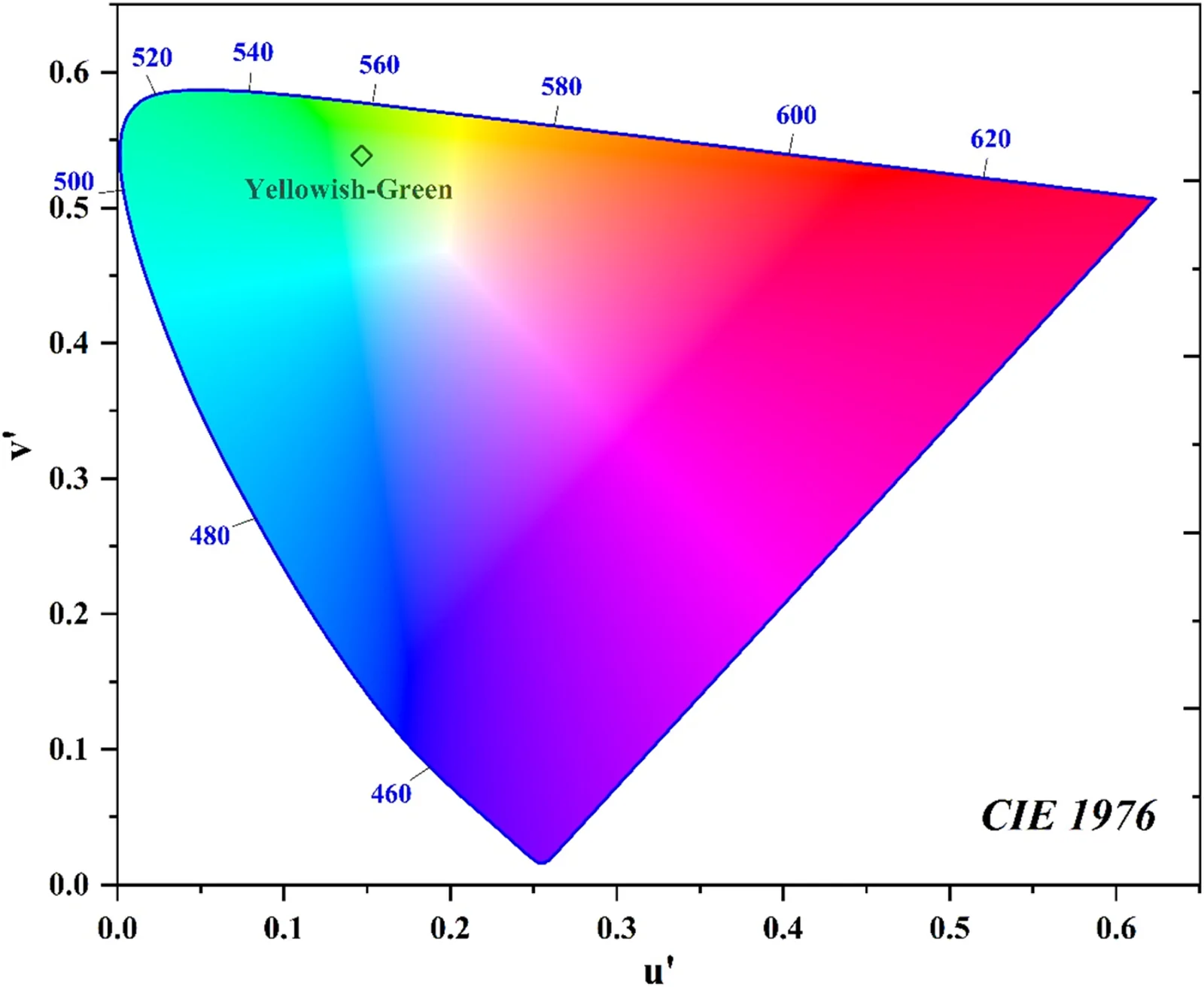
Chromaticity diagram CIE 1976.

Together, Fig. 15 and 16 establish that (C_5_H_6_N_3_O)_2_·[BiBr_5_] exhibits a direct and efficient phosphorescent emission, driven by SOC-enhanced excitonic relaxation and expressed as a spectrally pure yellow-green output. This behavior distinguishes the compound from typical purely organic emitters and situates it within the class of optically active Bi-based hybrids capable of supporting strong radiative transitions with technologically attractive colorimetric properties.

### 3.6. MEP, NCI–RDG, ELF, and LOL analyses

Fig. S7 presents the RDG/NCI surfaces and the corresponding RDG *vs.* sig *n*(*λ*_2_)*ρ* plot for (C_5_H_6_N_3_O)_2_·[BiBr_5_], visualizing the weak interactions governing supramolecular assembly. Strong hydrogen bonds (N1–H1B⋯N3 and N2–H2⋯O1) appear as compact blue basins with negative sig *n*(*λ*_2_)*ρ* values, indicating significant electron density accumulation consistent with short donor–acceptor distances and their classification as strong H-bonds. These interactions produce the dominant negative spikes in the RDG scatter, confirming their primary stabilizing role.

Weaker N–H⋯Br hydrogen bonds (N1–H1A⋯Br1 and N2–H2⋯Br1) appear as flattened green lobes, reflecting predominantly electrostatic attraction. In the RDG scatter, they correspond to small negative peaks near zero, indicating lower charge density and reduced directionality. Intra-plane Br⋯Br contacts, responsible for the pseudo-2D (*a*, *b*) packing, manifest as broad green sheets between neighboring bromides with near-zero sig *n*(*λ*_2_)*ρ* values, consistent with dispersion-driven interactions confined to individual planes. Smaller green regions highlight O⋯Br halogen–chalcogen contacts, which are weak but directional, refining the local packing around the organic cations.

Fig. S8 shows the MEP surface for a representative fragment of (C_5_H_6_N_3_O)_2_·[BiBr_5_], mapped onto an electron-density isosurface to reveal the polarity landscape governing intermolecular recognition. The most negative regions (red to orange) localize around the bromide termini of the BiBr_6_ unit, corresponding to experimentally observed N–H⋯Br acceptor sites and Br⋯Br dispersion sheets. The Bi center appears less negative due to polarization and its coordination environment, reflecting classical Bi–Br bonding anisotropy. Positive regions (blue) concentrate around protonated N1 and N2 atoms of the pyrazinamidinium cations, confirming their role as strong hydrogen-bond donors. Additional moderate positive regions near the amide hydrogen indicate weaker donor capacity, while the carbonyl oxygen (O1) forms a strong negative patch, consistent with its role as the primary hydrogen-bond acceptor (N2–H2⋯O1). The MEP highlights complementary organic–inorganic charge distribution, explaining directional N–H⋯Br interactions and the persistence of pseudo-2D Br⋯Br contacts.

Fig. S9 presents ELF and LOL maps for the same fragment, providing a topological view of electron localization across both BiBr_6_ and the pyrazinamidinium cations. The BiBr_6_ octahedron exhibits strong localization around Br ions, reflecting lone-pair density, with weak localization toward Bi, consistent with polarized ionic Bi–Br bonds. Absence of ELF basins at bond midpoints confirms the lack of covalent sharing. The asymmetric Br localization aligns with dispersion-driven Br⋯Br interactions seen in NCI maps.

Within the organic cations, ELF/LOL highlight well-defined covalent basins along C–N, C–C, and C–O bonds, with extended π-delocalization above and below the ring plane supporting π → π* transitions. Protonated N1 and N2 sites show localized ELF basins for N–H bonds and reduced lone-pair character, explaining strong positive MEP values and hydrogen-donor capacity, while O1 exhibits dense lone-pair localization, consistent with its acceptor role.

The spatial complementarity revealed by ELF/LOL explains experimental organic–inorganic interactions: electron-rich Br lobes align with polarized N–H donors, and localized carbonyl lone pairs engage in strong hydrogen bonding. Minimal ELF accumulation between fragments confirms noncovalent interface behavior governed by electrostatics and dispersion rather than orbital sharing. At the lattice level, ELF/LOL reinforce the MEP and RDG/NCI picture: the structure is stabilized by ionic Bi–Br bonds, localized organic π-framework covalency, and a hierarchy of hydrogen and halogen interactions, preserving one-dimensional BiBr_6_ chain integrity while enabling pseudo-2D supramolecular assembly.

## 4. Conclusion

This study presents a comprehensive investigation of the one-dimensional bismuth bromide hybrid (C_5_H_6_N_3_O)_2_·[BiBr_5_], incorporating protonated pyrazinamide cations. Single-crystal X-ray diffraction reveals a perovskite-related architecture composed of corner-sharing BiBr_6_ octahedra forming infinite chains. The organic cations contribute to crystal packing through N–H⋯Br and N–H⋯O hydrogen bonding, π–π stacking and additional intermolecular contacts. Hirshfeld surface analysis and fingerprint plots quantify the distribution of these interactions, while crystal void analysis indicates relatively compact packing, with a void volume of 8.22% of the unit-cell volume.

Vibrational spectroscopy supports the structural integrity of the organic and inorganic components and provides information on their bonding environments. Thermogravimetric analysis reveals a multistep decomposition process. Optical measurements indicate a direct optical transition with a reported band gap of approximately 2.8 eV, an Urbach energy of 0.175 eV and an absorption threshold near 456 nm. Photoluminescence measurements show a dominant emission band centered at 540 nm, corresponding to yellowish-green emission. The substantial Stokes shift and the strong spin–orbit coupling associated with Bi^3+^ are consistent with a possible contribution from relaxed triplet excited states; however, time-resolved measurements are required to establish the emission mechanism conclusively.

Computational analyses provide complementary insight into the electronic structure and intermolecular interactions. DOS/PDOS calculations indicate contributions from Br 4p states at the upper valence levels and Bi 6p and organic π* states among the accepting levels. The calculated molecular HOMO–LUMO gap is approximately 2.5 eV, although its comparison with the experimental optical gap must be interpreted in light of the finite-cluster approximation. ELF, LOL, MEP, and NCI–RDG analyses further characterize the electronic distribution and noncovalent interactions within the model. Overall, the results establish relationships between the crystal architecture, supramolecular organization and optical response of this pyrazinamide-based bismuth bromide hybrid, providing a basis for further investigation of low-dimensional, lead-free hybrid halides.

## Conflicts of interest

There are no conflicts to declare.

## Supplementary Material

RA-OLF-D6RA08608J-s001

## Data Availability

CCDC 2524117 contains the supplementary crystallographic data for this paper.^59^ The authors confirm that the data supporting the findings of this study are available within the article. Supplementary information (SI) is available. See DOI: https://doi.org/10.1039/d6ra08608j.
