# Supplementary material for: One-dimensional Bi–Br pyrazinamide-based hybrid halide perovskite: crystal architecture, intermolecular interactions, and structure–optical properties relationship
Source: RSC Adv. 2026 Nov 10. Online ahead of print. doi: 10.1039/d6ra08608j (PMC13750442; doi:10.1039/d6ra08608j)
Supplement: Supplementary file 1 [file RA-OLF-D6RA08608J-s001.pdf]

Supplementary Information (SI) for RSC Advances.
This journal is © The Royal Society of Chemistry

Supplementary

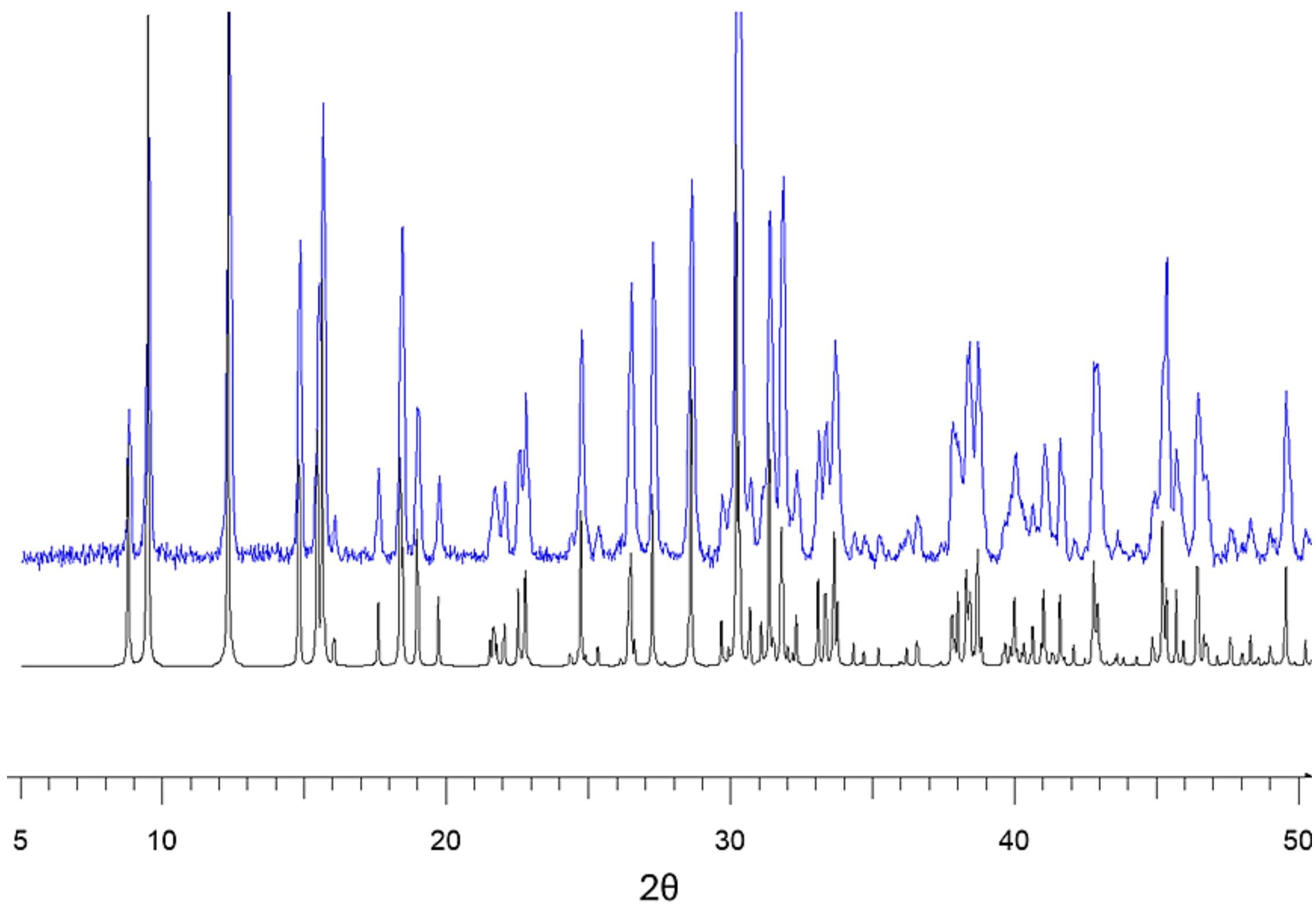

Fig. 1.S: Experimental (blue) vs calculated (black) peaks of PXRD

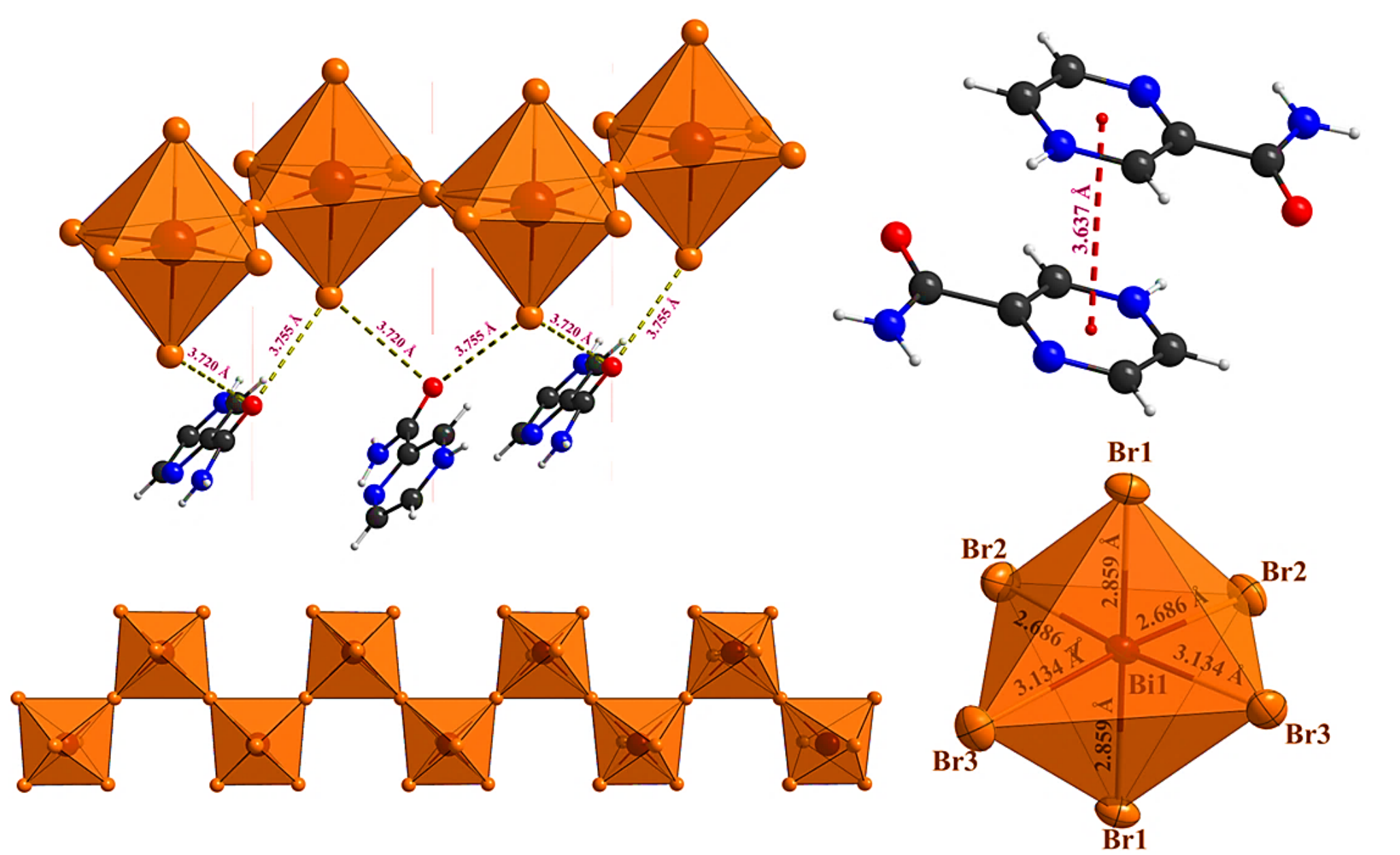

Fig. 2.S: Polyhedral features, secondary interactions, and comparative structural context

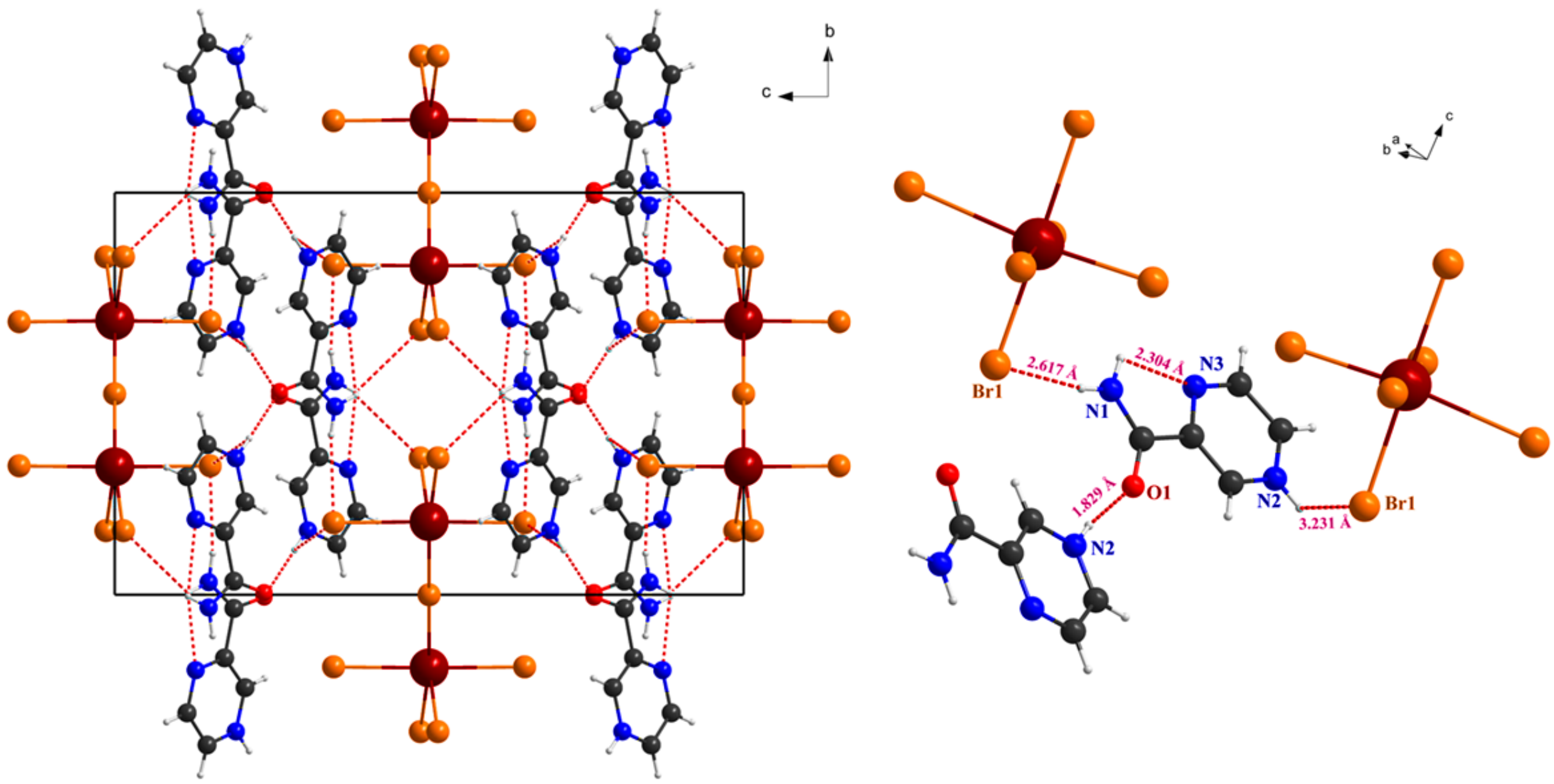

Fig. 3.S: Hydrogen-bonding architecture and three-dimensional reinforcement

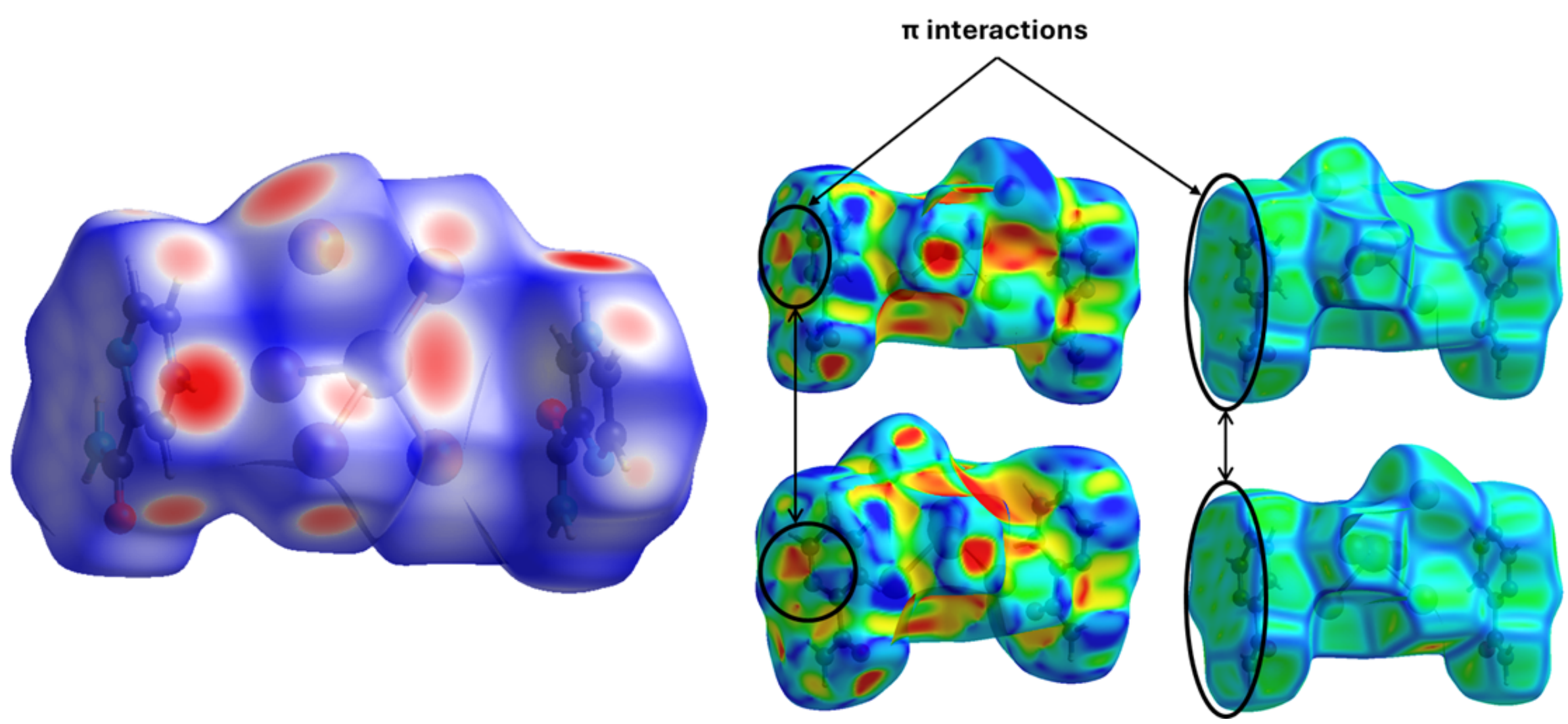

Fig. 4.S: Hirshfeld surface of the titled: **(a)** 3D dnorm surface, **(b)** surface index, **(c)** curvedness

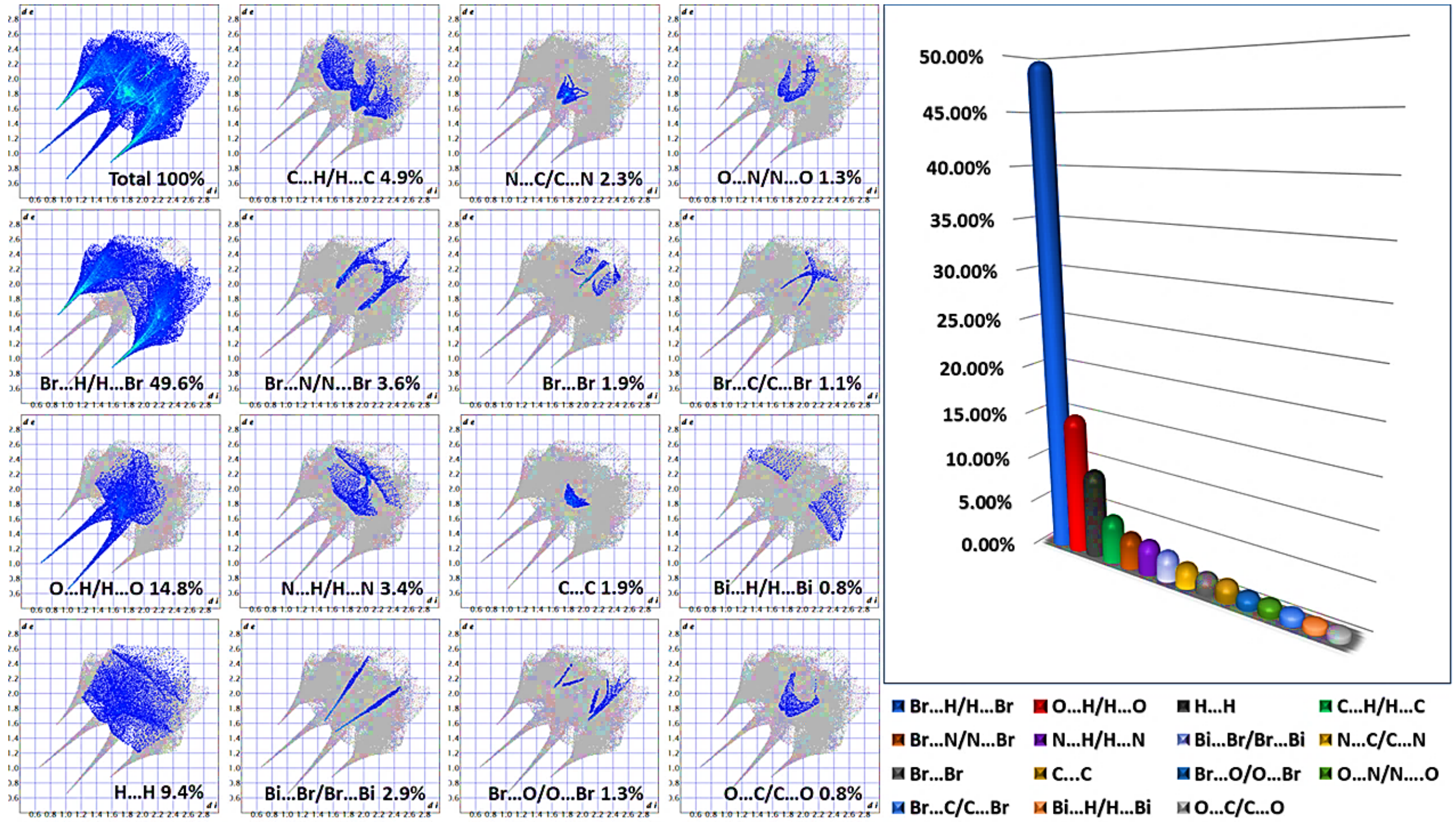

Fig. 5.S: 2D Visualization of Total Contact Contributions to the Hirshfeld Surface

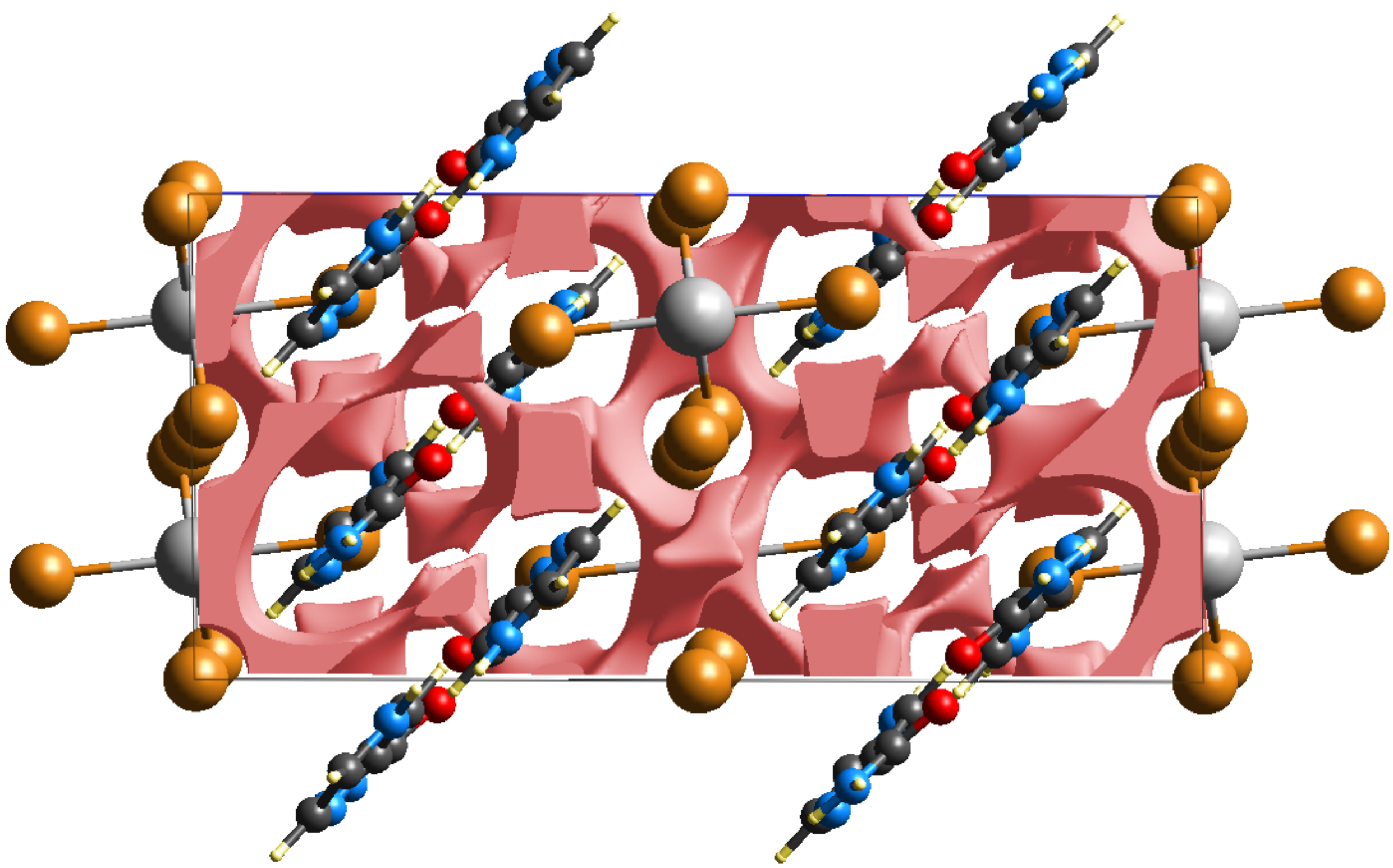

Fig. 6.S: Presence of a Crystal Void in the Compound

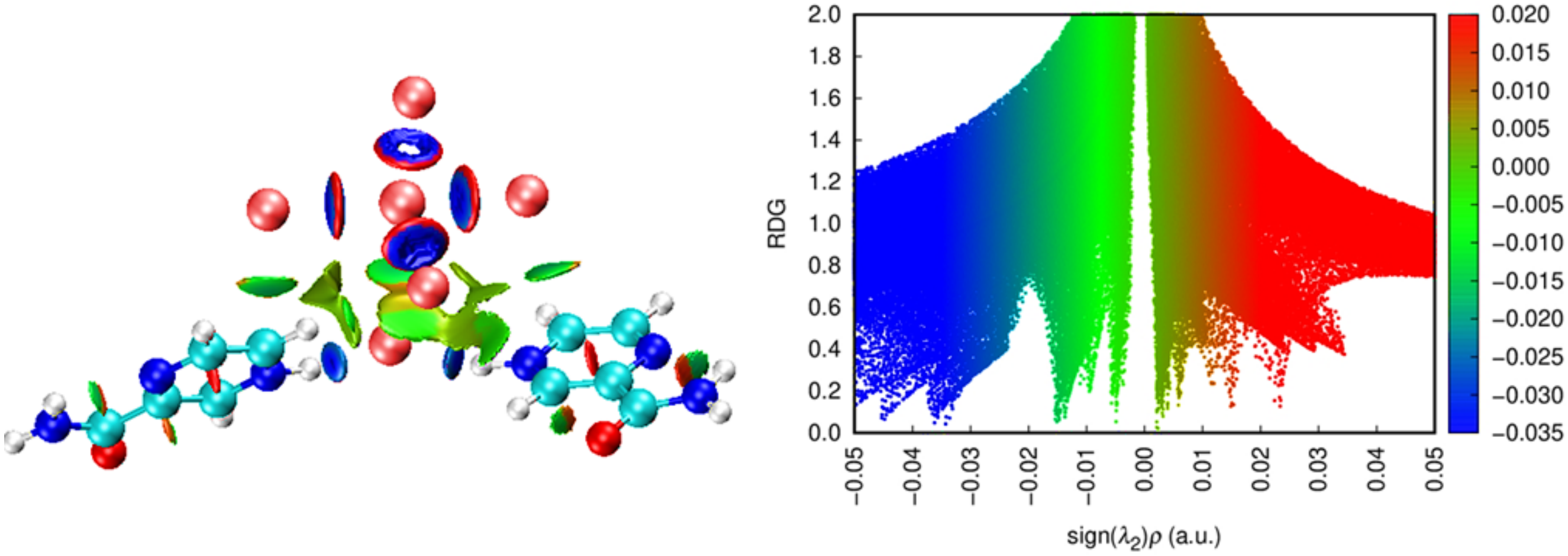

(a) **(b)**

Fig. 7.S: (a) NCI and (b) RDG Visualization of the Target Compound

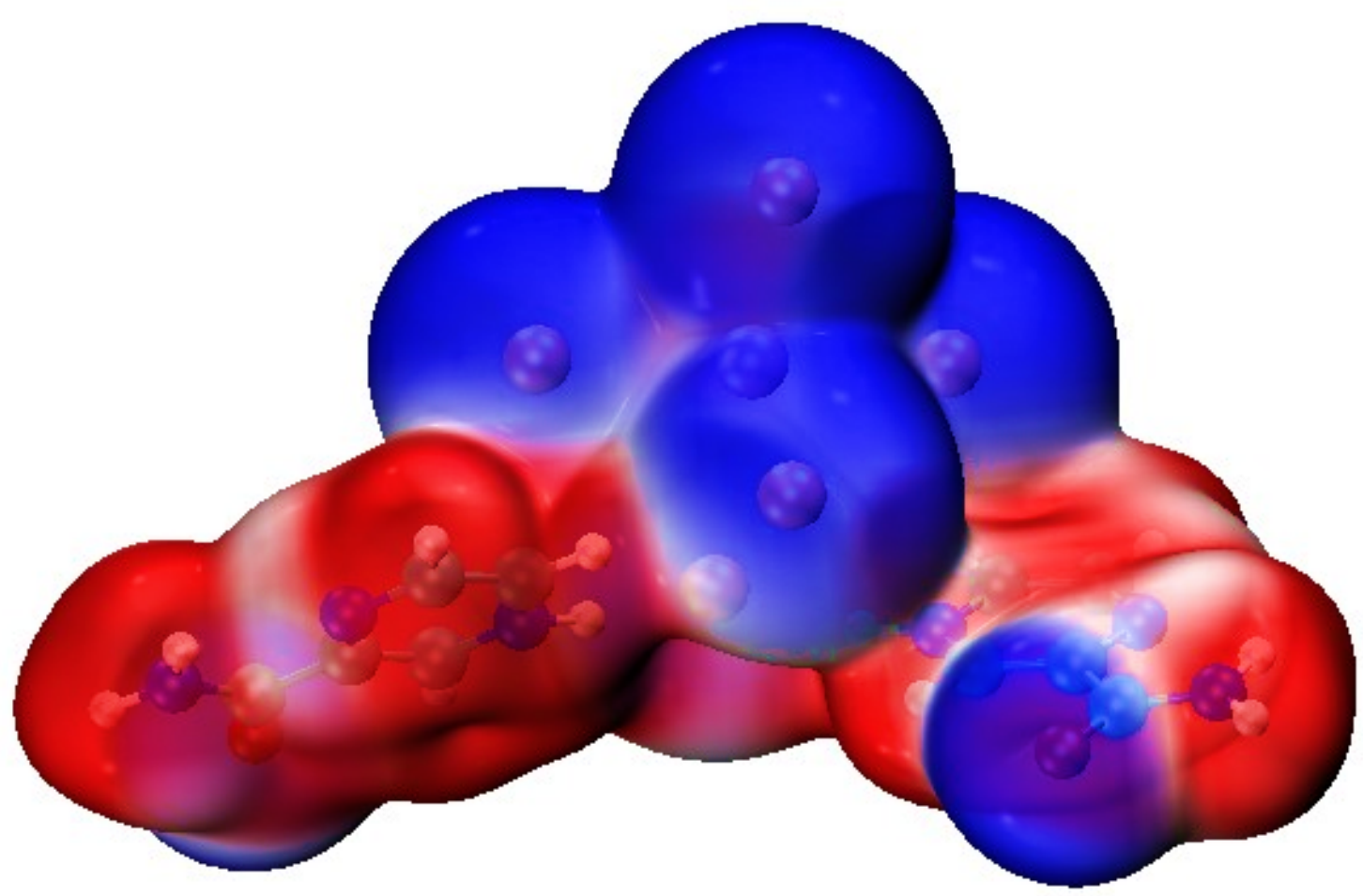

Fig. 8.S: Molecular Electrostatic Potential Analysis of the Target Compound

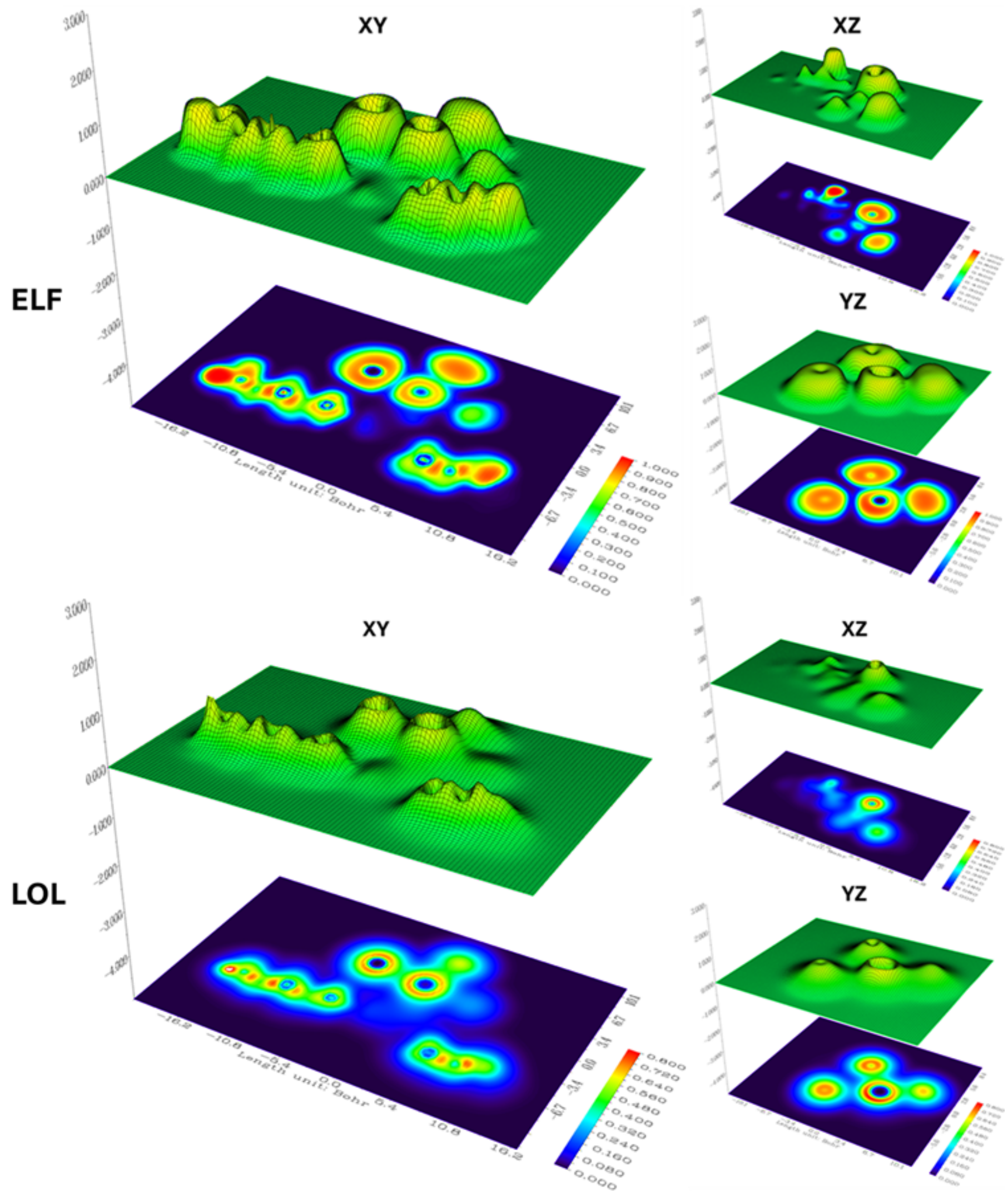

Fig. 9.S: Electron Localization Function (ELF) and LOL visualization in (XY), (XZ), and (YZ) planes

Table 1.S: Summary of crystal data and structure refinement details

Crystallographic data	
**Empirical Formula**	$(C_5H_6N_3O)_2\cdot[BiBr_5]$
**Color/Shape**	Yellow / Prism
**Molar mass (g/mol)**	856.79
**Diffractometer**	XtaLAB Synergy, Dualflex, HyPix diffractometer
**Radiation type**	Mo *Kα* (0.71073 Å)
**Absorption Correction**	Multi-scan
**Crystal system**	Monoclinic
**Space group**	I2/a
**Z / Z'**	4 / 0.5
**Unit Cell Parameters**	

a (Å)	9.0086 (5)
b (Å)	11.9836 (6)
c (Å)	18.7178 (10)
□ (°)	90.870 (2)
Absorption coefficient (mm^{-1})	18.63
Crystal size $[mm]^3$	0.15 × 0.15 × 0.05
Number of reflections measured Variation of h, k, l	h= -10 → 10, k= -14 → 11, l= –22 → 16
Scanning range of θ(°)	2.8 < □ < 25.0
Number of measured, independent, and observed [I > 2σ(I)] reflections	5079 / 1784 / 1474
F(000)	1552
Independent Parameters	111
$□□_{max}$ / $□□_{min}$ (e $Å^{-3}$)<br>$(Δ/σ)_{max}$	1.28 / –1.66<br>< 0.001
$R[F^2 > 2σ(F^2)] = R_1$	0.039
$wR(F^2) = wR_2$	0.068
S = GooF	1.02
CCDC	**2524117**

Table 2.S: Comparison of selected one-dimensional BiBr₅-based organic–inorganic hybrid halides

Compound (reference)	Inorganic structure	Reported optical/functional properties	Distinction from the present compound
(C₅H₆N₃O)₂[BiBr₅] (this work)	• 1D corner-sharing BiBr₆ chains<br>• Monoclinic I2/a<br>• Pyrazinamidinium cations	• Direct optical gap: ~2.8 eV<br>• Urbach energy: 0.175 eV<br>• PL maximum: 540 nm	• Multifunctional aromatic amide cation<br>• Combined SCXRD, Hirshfeld, optical and computational analyses
(TMBD)BiBr₅ [55]	• 1D corner-sharing chains<br>• Near-regular BiBr₆ octahedra<br>• Di-tertiary ammonium spacer	• Photodetector responsivity: 82.97 mA W⁻¹<br>• Detectivity: 8.06 10¹¹ Jones<br>• Suppressed ion migration	• Emphasis on charge transport and photodetection rather than detailed supramolecular and optical characterization.
(C₂H₅NH₃)₂[BiBr₅] [56]	• 1D polymeric $[BiBr_5]_n^{2n-}$ chains<br>• Ethylammonium cations<br>• Temperature-dependent structural phases	• Optical gap: ~2.6 eV<br>• Ferroelectric phases below 160 K<br>• Reversible phase transitions	• Ferroelectric and phase-transition behavior rather than aromatic-cation-directed packing.
(C₆H₁₄N)₂[BiBr₅] [57]	• 1D zigzag chains<br>• BiBr₆ octahedra sharing two cis-Br atoms<br>• Monoclinic $P2_1/c$	• Blue PL emission: 2.71 eV<br>• Absorption peaks: 3.01, 3.73 and 4.4 eV<br>• DFT and vibrational analyses	• Edge-sharing-type connectivity and cyclohexylammonium cations, unlike the single-corner-sharing chain in the present material.

(H₂DMAPA)BiBr₅ [58]	• 1D zigzag chains<br>• BiBr₆ octahedra sharing two adjacent Br atoms<br>• Monoclinic $P2_1/n$	• Optical gap: 2.65 eV<br>• PL maximum: 480 nm<br>• Excitation wavelength: 440 nm	• Different diammonium spacer and chain connectivity; emphasis on cation-dependent optical tuning.

Table 3.S: The attributions of calculated and observed frequencies of the vibration modes of the compound

IR (cm^{-1})	Raman (cm^{-1})	Calc. wavenumbers (cm^{-1})	Assignment
3406	-	3401	υ$_{as}$(NH_2)
3271	-	3242	υ$_s$(NH_2)
3102	-	3047	υ$_{as}$(C-H)
3048	-	3001	υ$_s$(C-H)
2819	-	2797	υ(N-H)
1950-2750	-	2724	υ(N-H...Br)
1673	-	1699	υ(C=O)
1608	-	1625	υ(C=N)
1518	-	1535	υ(C=C)
1444	-	1427	δ(NH_2)
1315	-	1338	υ(C-N) Amide
1276	-	1297	υ(C-N)
1247	-	1276	δ(C-H)
1155	-	1169	β(C-H)
1138	-	1121	υ(C-C)
1069	-	1060	ρ(NH_2)
979	-	987	γ(C-H)
733	-	780	ω(NH_2)
672	-	680	δ(C=C-N)
576	-	603	δ(C=N-C)
-	157	165	υ$_{as}$(Bi-Br)
-	88	106	δ (Br-Bi-Br)
-	64	57	Lattice Mode

υ$_s$:symetric stretching, υ$_{as}$:assymetric stretching, β : in plane bending, γ :out plane bending, δ :scissoring, ρ : rocking, ω:wagging, τ : Twisting
